# Spontaneous and Laccase‐Assisted Adsorption of Hemicelluloses Rendering Colloidal Stability of Lignin Nanoparticles at Low pH

**DOI:** 10.1002/cssc.202500668

**Published:** 2025-05-30

**Authors:** Patrícia Figueiredo, Danila Morais de Carvalho, Kristiina S. Hilden, Maarit H. Lahtinen, Kirsi S. Mikkonen

**Affiliations:** ^1^ Department of Food and Nutrition Faculty of Agriculture and Forestry University of Helsinki P.O. Box 66 FI‐00014 Helsinki Finland; ^2^ Department of Microbiology Faculty of Agriculture and Forestry University of Helsinki FI‐00014 Helsinki Finland; ^3^ Helsinki Institute of Sustainability Science (HELSUS) University of Helsinki P.O. Box 65 FI‐00014 Helsinki Finland

**Keywords:** hardwood, hemicelluloses, laccases, lignin nanoparticles, softwood

## Abstract

Functionalizing lignin nanoparticles (LNPs) is an attractive strategy to improve their properties and expand their range of applications. However, conventional approaches to achieve such modifications are done using environmentally and energy‐demanding methods. Here, a green synthetic approach through spontaneous adsorption and laccase‐induced reactions to functionalize LNPs with hemicelluloses is introduced. Structurally varying lignin and hemicellulose grades isolated from different sources and several fungal laccases are systematically compared. Specifically, the residual lignin content in hemicelluloses is hypothesized to determine their adsorption efficacy on LNPs. Results reveal that the LNPs can be coated with hemicelluloses by physical adsorption, which greatly increases their colloidal stability at acidic pH, compared to plain LNPs. Furthermore, fungal laccases can play an active role in attaching lignin moieties of the hemicelluloses at the LNP surface, confirmed by the enriched lignin‐derived compounds and monosaccharide composition of the laccase‐treated hybrid LNPs, compared to the physical adsorption of hemicellulose on the LNP surface. Overall, this study provides new insights on the mechanism of laccase‐assisted functionalization of LNPs with other ligands (e.g., hemicelluloses). Furthermore, the increased colloidal stability of LNPs can greatly increase the application of such hemicellulose–lignin hybrid nanosystems for different areas, such as health sciences.

## Graphical abstract

Here, a green synthetic approach through spontaneous adsorption and laccase‐induced reactions is introduced to functionalize lignin nanoparticles (LNPs) with hemicelluloses. The resulting lignin–hemicellulose hybrid nanoparticles presented increase colloidal stability at acidic pH compared to the plain LNPs, encouraging their usage in different areas, such as food sciences and oral drug delivery.

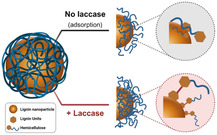

## 1 Introduction

Lignin is one of the most abundant biopolymers and a polyphenolic macromolecule, constituted mainly by p‐hydroxyphenyl (H), guaiacyl (G), and syringyl (S) units interconnected by aryl ether and carbon–carbon bonds, and the proportion of these units varies according to the extraction method and source of plant biomass.^[^ ^1^, ^2^, ^3^, ^4^ ^]^ Lignin is a very interesting polymer for a variety of applications due to its unique and intrinsic properties, such as biodegradability and biocompatibility, antioxidant, UV‐blocking, and antimicrobial abilities.^[^ ^4^, ^5^ ^]^ However, the complex and heterogeneous molecular structure of the lignin polymer can hamper its valorization, and therefore, the production of lignin nanoparticles (LNPs) can be done to control the morphology and improve the properties of lignin.^[^ ^6^, ^7^ ^]^ In addition, the transformation of the typically water‐insoluble lignin into stable LNP colloidal dispersions in water results in a higher specific surface area with improved antioxidant activity.^[^ ^4^, ^8^, ^9^ ^]^ The LNP surface can be further modified and functionalized, as a result of the large availability of different functional groups, including aliphatic and phenolic hydroxyl and carboxyl groups.^[^ ^4^, ^10^, ^11^ ^]^ Different modification approaches to functionalize the LNP surface have been conducted to improve the targeted and controlled release of active compounds loaded into the LNPs, stabilizing the LNP surface against certain solvents and extreme pH conditions.^[^ ^8^, ^10^, ^12^, ^13^ ^]^

Enzymatic treatment of lignocellulosic materials has attracted increased attention due to their cost‐effective, sustainable, and environmentally friendly approaches compared to the conventional methodologies, being employed for a variety of applications such as lignin breakdown, bioremediation and environmental protection, as well as controlled release of fungicides in agriculture.^[^ ^14^, ^15^, ^16^, ^17^ ^]^ In particular, phenol‐oxidizing metalloenzymes such as laccases can act on polymeric aromatic and phenolic compounds (e.g., lignin), being able to polymerize or depolymerize lignin and phenols depending on the reaction conditions, such as the pH, dosage of the laccase, and type of lignin substrate.^[^ ^15^, ^18^, ^19^ ^]^ Laccases can promote the lignin depolymerization, which is accompanied by the increase in the phenolic hydroxyl content and decrease in molar mass after treatment. On the other hand, laccases can generate phenoxy radicals from phenolic hydroxyl groups that can undergo both oxidative coupling, leading to lignin polymerization, or lignin oxidation at the Cα position.^[^ ^20^, ^21^ ^]^ For example, Mattinen et al. demonstrated that laccase treatment of softwood‐based LNPs led to covalent intraparticle crosslinking without significant changes in particle size, making them stable in organic solvents.^[^ ^22^ ^]^ Moreover, we have previously studied the mechanism of LNPs’ oxidation/polymerization after treatment with fungal laccases under acidic conditions, where we observed an increase in the particle size of the LNPs with low S/G ratio (softwood lignin) after laccase treatment, suggesting the polymerization and crosslinking of the LNPs.^[^ ^23^ ^]^ On the other hand, the LNPs presenting a high S/G ratio (hardwood lignin) showed a decrease in the phenolic content together with an increase in the percentage of oxidized S‐type units, indicating a preferred Cα–OH type of oxidation.

In this study, we propose to functionalize the LNPs with hemicelluloses, either by spontaneous adsorption or laccase‐assisted reaction, to increase the colloidal stability of the LNPs at acidic pH. With this approach, our hemicellulose–lignin hybrid nanosystems could serve as efficient carriers to encapsulate and improve the performance of nutraceutical and therapeutic compounds after oral administration. These hybrid nanoplatforms could prevent the degradation and premature release of their cargoes in the acidic gastric environment and improve their stability, aqueous solubility, bioavailability, and absorption in the intestine.^[^ ^24^, ^25^ ^]^ Additionally, natural hemicelluloses can also exhibit mucoadhesive properties due to the presence of xyloglucan or hyperbranched structure (e.g., arabinogalactan), which shows greater adhesion ascribed to higher surface free energy and more exposed hydroxyl groups, prolonging the nanoparticle adhesion to mucosal surfaces.^[^ ^26^, ^27^ ^]^ Hemicelluloses are a family of polysaccharides that are also extracted from plant biomass, formed by different derivatives of pyranose and furanose sugar units.^[^ ^28^ ^]^ The structural composition of hemicelluloses is highly dependent on the source of the wood biomass from where the hemicelluloses are isolated: softwoods are typically rich in galactoglucomannan (GGM, 16%–17% of dry wood weight) with some arabinoglucuronoxylan, while hardwoods are rich in glucuronoxylan (GX, 15%–30%).^[^ ^28^, ^29^, ^30^ ^]^ Depending on the isolation process, the molar mass distribution and solubility of the typically water‐soluble hemicelluloses are affected by their purity, being that the hemicelluloses are chemically connected to lignin to form lignin–carbohydrate complexes (LCCs) via phenylglycoside, benzylether, and gamma–ester bonds.^[^ ^29^, ^31^, ^32^ ^]^ Here, we selected two hemicelluloses isolated from different sources (GGM and GX), containing residual lignin, to evaluate if the source and composition of the hemicellulose can have an impact on the efficacy of the LNP coating. In addition, both hemicelluloses were submitted to an ethanol precipitation treatment to reduce the lignin content in their structures and to study the influence of the lignin content of hemicelluloses on the LNP coating. Therefore, the main aim of this study is to find the optimal conditions for an effective functionalization of LNPs with hemicelluloses, to improve their colloidal stability at acidic pH compared to the bare LNPs that aggregate at acidic pH. The LNPs were first prepared by antisolvent precipitation using three different technical lignins as starting material, incubated with hemicelluloses by spontaneous adsorption or in the presence of fungal laccases, and further characterized in terms of their physicochemical properties and stability at acidic pH. Our results show that the functionalization of LNPs with hemicelluloses is more effective when lignin‐richer hemicelluloses were used for the reaction, in the presence of laccases. Therefore, the laccase treatment represents a promising tool in the development of lignin–hemicellulose hybrid nanosystems for upcoming bioproducts.

## 2 Results and Discussion

### 2.1 Physicochemical Characterization of Bare LNPs

The antisolvent precipitation approach is commonly used to achieve spherical LNPs, with uniform size and high colloidal stability, which greatly increases the use of such nanoplatforms for health sciences. We have previously reported that the acetone/water (3:1) mixture as the lignin solvent, and water as the anti‐solvent, was the optimal approach to prepare the spherical LNPs from different lignin grades.^[^ ^33^ ^]^ Here, we have used this approach to obtain the LNPs from three technical lignins as starting polymers: softwood kraft LignoBoost (LB), wheat straw/Sarkanda grass alkali Protobind 1000 (PB), and hardwood BLN birch lignin (BB), rendering LB‐LNPs, PB‐LNPs, and BB‐LNPs, respectively. The as‐prepared LNPs were characterized by dynamic light scattering (DLS) for their average size, polydispersity index (PDI), and ζ‐potential, and transmission electron microscopy (TEM) for their morphology. In addition, the amounts of phenolic units were assessed by pyrolysis gas chromatography mass spectrometry (Py‐GCMS), and the presence of functional groups on the LNP surface was evaluated using phosphorus‐31 nuclear magnetic resonance (^31^P‐NMR) (**Table** **1**).

**Table 1:** Characterization of LB‐, PB‐, and BB‐LNPs in terms of their average size, PDI, and ζ‐potential using DLS (n ≥ 3) and ^31^P‐NMR.

	LB‐LNPs	PB‐LNPs	BB‐LNPs
TEM			
	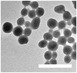	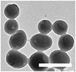	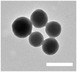
DLS			
Size (nm)	116 ± 5	170 ± 5	181 ± 4
PDI	0.148 ± 0.006	0.119 ± 0.007	0.121 ± 0.003
ζ‐potential (mV)	−44.9 ± 1.3	−47.6 ± 0.6	−48.0 ± 0.4
Py‐GCMS			
Total G (%)	88.4	44.0	29.7
Total S (%)	–	44.2	63.4
Other Aromatics (%)	7.9	10.3	6.9
S/G ratio	0.0	1.0	2.1
Sulfur (%)	3.7	1.5	–
^31^P NMR (mmol/g)			
COOH	0.35	0.64	0.62
Phenolic OH	3.46	2.62	3.33
Aliphatic OH	2.26	1.21	1.14

Normalized area of the main lignin‐derived monolignols (G, guaiacyl; S, syringyl) identified using Py‐GCMS. TEM pictures of the LNPs (scale bars = 200 nm).

Abbreviations: BB, hardwood; BLN birch lignin; DLS, dynamic light scattering; LB, softwood kraft LignoBoost; LNPs, lignin nanoparticles; PB, wheat straw/Sarkanda grass alkali Protobind 1000; PDI, polydispersity index; ^31^P‐NMR, phosphorus‐31 nuclear magnetic resonance; Py‐GCMS, pyrolysis gas chromatography mass spectrometry; TEM, transmission electron microscopy.

The hydrodynamic diameter of LNPs varied according to the molar mass of the technical lignin (LB > PB ≥ BB), that is, the higher the molar mass, the lower the LNP size,^[^ ^33^ ^]^ due to the faster nucleation kinetics in high‐molar‐mass lignin that gives rise to smaller LNPs. Therefore, the LB‐LNPs exhibited a slightly smaller size than that of the PB‐ and BB‐LNPs, which can also be attributed to other factors, such as: 1) stronger hydrophobic interactions in LB compared to the other two lignins that affect the nucleation kinetics during the LNP preparation, that is, more hydrophobic lignin molecules result in smaller LNPs; 2) a higher amount of aliphatic hydroxyl groups in LB than in PB and BB lignins (as measured by ^31^P‐NMR) can result in the formation of smaller LNPs, due to the reduced intermolecular bonding between lignin molecules; 3) the chemical composition of lignin, that is, higher percentage of G units on LB structure, can lead to stronger noncovalent π–π interactions between G units than the interactions between the S units on both PB and BB lignins, resulting in a denser packing of lignin molecules during LNP formation and consequently, to the smaller average size of LB‐LNPs; 4) molecular conformation and higher aromatic density of LB molecules can affect the packing density of the resulting LNPs, that is, the branched substructure and stiffness of LB molecules due to the condensed C—C bonds between aromatic rings may confer a denser core packing, yielding smaller LNPs.^[^ ^9^, ^34^, ^35^, ^36^ ^]^

The TEM pictures showed the formation of spherical LNPs and also confirmed the trend on the size of the prepared LNPs. All the LNPs exhibited PDI values lower than 0.15, indicating that the LNPs are homogeneous and monodispersed. Nevertheless, the LB‐LNPs presented the highest PDI value, which can be ascribed to the higher heterogeneity of the kraft lignin compared to the other technical lignins. The ζ‐potential of LNPs was negative due to the presence of carboxyl groups on the LNP surface, which leads to the stabilization of the LNPs in colloidal suspensions due to the electric double‐layer repulsion. Furthermore, both PB‐ and BB‐LNPs presented a more negative surface charge than the LB‐LNPs (≈−48 vs. −44 mV), due to the higher amount of —COOH groups in their surface, as observed by ^31^P‐NMR.

Py‐GCMS was then used to evaluate the percentage of monolignols and S/G ratio in the LNPs obtained from the different technical lignins, which can have an impact on the laccase efficiency for possible crosslinking and functionalization of LNPs with other ligands. We observed that the softwood LB‐LNPs exhibited the highest amount of G units (≈88%), while the PB‐ and BB‐LNPs showed about 44% and 30%, respectively. On the other hand, the hardwood BB‐LNPs and the Sarkanda grass‐derived PB‐LNPs exhibited 63% and 44% of S units, respectively, whereas the LB‐LNPs did not present S units in their composition. Consequently, the S/G ratio in BB‐LNPs (2.1) was substantially higher than in PB‐LNPs (1.0) and LB‐LNPs (0.0). Furthermore, we detected some sulfur‐derived compounds after pyrolytic breakdown of LB‐ and PB‐LNPs even though sulfur is not a natural component in the lignin structure, which can be ascribed to the extraction and cooking process applied to isolate the lignin, in particular during the kraft pulping process.^[^ ^37^ ^]^ Furthermore, the fractionation of the technical lignins during the preparation of LNPs did not affect the chemical composition and proportion of the lignin units (Figure S1a, Supporting Information), with S/G ratios for technical BB, PB, and LB lignins of 2.5, 1.1, and 0.0, respectively. In addition, we also quantified the monosaccharides in the technical lignins and respective LNPs (Figure S1b and Table S1, Supporting Information). We observed that softwood LB and Sarkanda grass PB lignins contained more carbohydrates than the hardwood BB lignin, and the proportion of all carbohydrates was reduced after LNP preparation compared to the technical lignins. This might be due to the fact that the technical lignins are initially dissolved in the acetone/water mixture for the LNP preparation, and the hemicelluloses are not soluble in organic solvents. After the filtration step, the acetone‐insoluble fraction of lignin sample that can contain some hemicellulose portion is removed, and therefore, the total percentage of carbohydrates decreased after LNP preparation from 3.1%–4.3% to 1.2%–2.7%.

### 2.2 Characterization of the Lignin–Hemicellulose Hybrid NPs After Spontaneous Adsorption and Laccase‐Assisted Reaction of Hemicelluloses with LNPs

**Figure 1:**
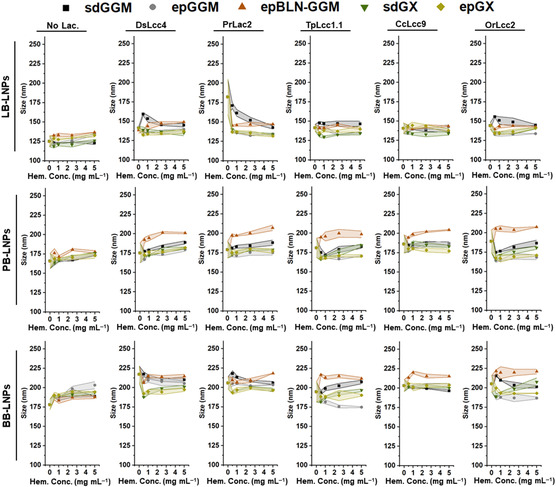
Size of the LNPs measured by dynamic light scattering after incubation of LNPs with the different laccases (1000 nKat g^−1^ of lignin) and hemicelluloses (0.5–5 mg mL^−1^), at pH 5 and RT for 24 h. Control samples were also analyzed in the absence of laccase and/or hemicelluloses. The lines between the symbols are to guide the eye, and their width represents the s.d. of each measured point (n ≥ 3). Abbreviations: BB, hardwood BLN birch lignin; ep, ethanol precipitated; epBLN‐GGM, ethanol‐precipitated galactoglucomannan isolated using the BLN process; GGM, galactoglucomannan; GX, glucuronoxylan; LB, softwood kraft LignoBoost; LNPs, lignin nanoparticles; PB, wheat straw/Sarkanda grass alkali Protobind 1000; sd, spray dried.

The surface modification or functionalization of LNPs can further improve the properties of LNPs and increase their range of applications. This functionalization can be done through covalent and noncovalent modification of LNPs, by grafting targeting ligands that can increase the LNP uptake by specific cells/tissues and/or polymers to improve the stability and integrity of LNPs against extreme solvent and pH.^[^ ^12^, ^13^, ^38^ ^]^ Here, we aimed to find the optimal conditions for functionalizing the LNPs with hemicelluloses, which were isolated from both softwood (spray‐dried (sd) GGM and ethanol‐precipitated (ep) BLN‐GGM) and hardwood (sdGX), and the sd‐hemicelluloses were submitted to ethanol precipitation treatment to decrease the lignin content in their compositions. The composition and characteristics of the hemicellulose are detailed in Table S2, Supporting Information. The lignin content in the hemicelluloses, determined by Py‐GCMS, follows this trend: epBLN‐GGM < epGGM < epGX < sdGGM < sdGX. In order to assess the impact of the lignin content of hemicelluloses and their concentration on the LNP functionalization, we have reacted the hemicelluloses (0.5–5 mg mL^**−1**^) with 1 mg mL^**−1**^ of LNPs, by spontaneous adsorption and assisted by laccase treatments (1000 nKat g^**−1**^ of lignin). Initially, the size of LNPs was measured to evaluate the effect of the hemicellulose coating with and without the laccase treatment (**Figure** **1**).

For softwood LB‐LNPs, the size of the LNPs that were not treated with laccases remained similar or slightly higher as the hemicellulose concentration in the reaction increased. On the other hand, the laccase‐treated LB‐LNPs experienced a slight increase in particle size compared with non‐treated LNPs, which could be ascribed to the inter‐crosslinking of particles/lignin due to the formation of oxidized dimeric products derived from the polymerization of G‐type‐rich softwood LNPs.^[^ ^23^ ^]^ However, after adding the hemicelluloses in the reaction, the average size of LNPs slightly decreased with the increase in hemicellulose concentration, which might indicate that the hemicellulose coating can improve colloidal stability of LNPs possibly due to the steric repulsion between lignin‐hemicellulose hybrid NPs. As for the PB‐ and BB‐LNPs, their size after laccase treatment did not increase substantially, due to the higher content on S‐type units that have the 5‐position on the aromatic ring occupied by the methoxy group, preventing the LNP polymerization. In addition, the size of the PB‐ and BB‐LNPs experienced a slight increase after adding the hemicelluloses at different concentrations, with values ranging between 180 and 225 nm. The long‐term stability of lignin–hemicellulose hybrid NPs was monitored, and the LNP size remained similar after 7 weeks of storage at 4 °C (Figure S2, Supporting Information). Furthermore, the PDI of the LNPs was lower than 0.2, indicating the presence of monodispersed LNP suspensions (Figure S3, Supporting Information). The PDI values remained similar or even decreased after functionalization of LNPs with hemicelluloses, also suggesting that the hemicellulose coating can stabilize the LNPs. In addition, the morphology of the LNPs did not change after laccase treatments and/or hemicellulose coating, as observed in the representative images in Figure S4, Supporting Information.

**Figure 2:**
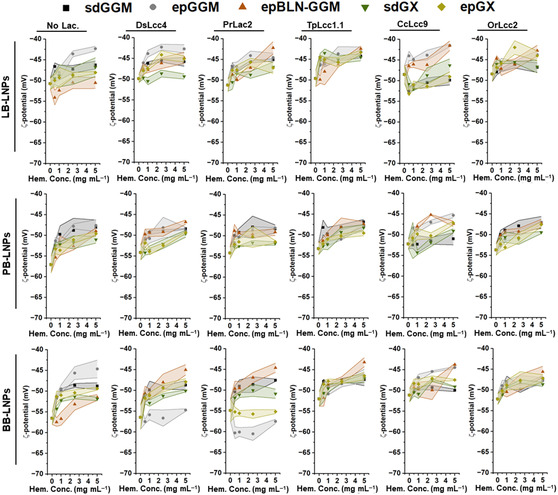
Surface charge of LNPs, given by the ζ‐potential, measured by dynamic light scattering after incubation of LNPs with the different laccases (1000 nKat g^−1^ of lignin) and hemicelluloses (0.5–5 mg mL^−1^), at pH 5 and RT for 24 h. Control samples were also analyzed in the absence of laccase and/or hemicelluloses. The lines between the symbols are to guide the eye, and their width represents the s.d. of each measured point (n ≥ 3). Abbreviations: BB, hardwood BLN birch lignin; ep, ethanol precipitated; epBLN‐GGM, ethanol‐precipitated galactoglucomannan isolated using the BLN process; GGM, galactoglucomannan; GX, glucuronoxylan; LB, softwood kraft LignoBoost; LNPs, lignin nanoparticles; PB, wheat straw/Sarkanda grass alkali Protobind 1000; sd, spray dried.

The surface charge of LNPs, here given by their ζ‐potential values, was quantified to evaluate the effect of the concentration and type of hemicellulose on the LNP coating after reaction (**Figure** **2**). As expected, the ζ‐potential values increased as the hemicellulose concentration in the reaction increased, from ≈57 up to −42 mV, which indicates that the hemicellulose coating was successful. Generally, the ζ‐potential of the ethanol‐precipitated GGMs and GX samples was slightly higher than the sdGGM/GX, due to the lower lignin content that gives to the ethanol‐precipitated hemicelluloses a slightly higher ζ‐potential than the respective sdGGM/GX (Table S2, Supporting Information). In addition, the trends on the ζ‐potential of LNPs remained similar after 7 weeks of storage at 4 °C, suggesting a good long‐term stability of the hemicellulose‐coated LNPs (Figure S5, Supporting Information).

### 2.3 Colloidal Stability of the Lignin–Hemicellulose Hybrid NPs Suspensions

The colloidal stability of nanoparticles is affected by their surface properties, including the presence of functional groups, surface charge, and morphology, which in turns will influence their biological response and viability for further applications.^[^ ^39^, ^40^ ^]^ Here, we first evaluated the colloidal stability of LNPs in MQ‐water using Turbiscan, by measuring the variation of the light transmitted (ΔT) through the LNP suspensions at 0.5 mg mL^−1^ up to 30 days (Figure S6, Supporting Information). The percentage of ΔT varied according to the size of the LNPs, that is, the lower the LNP size, the lower the ΔT, and consequently, the higher the stability. Therefore, the colloidal stability of LNPs varied according to the following order: LB‐LNPs (ΔT up to 17%; 116 nm) > PB‐LNPs (ΔT up to 30%; 170 nm) > BB‐LNPs (ΔT up to 40%; 180 nm). In addition, the ΔT was slightly lower after hemicellulose coating of LNPs, in particular for LB‐LNPs, suggesting a higher stability of the hemicellulose‐functionalized LNPs than the bare LNPs. Finally, the LNPs could be easily redispersed after 30 days of storage for further applications.

**Figure 3:**
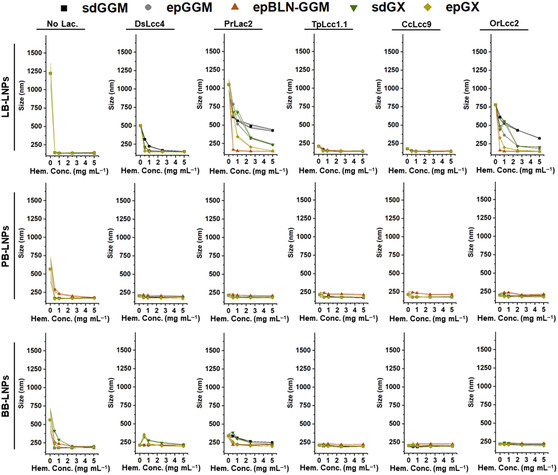
Stability of LNPs suspensions in 25 mm citric acid pH 3 by dynamic light scattering in terms of their size, after incubation with the different laccases (1000 nKat g^−1^ of lignin) and hemicelluloses (0.5–5 mg mL^−1^), at pH 5 and RT for 24 h. Control samples were also analyzed in the absence of laccase and/or hemicelluloses. The lines between the symbols are to guide the eye, and their width represents the s.d. of each measured point (n ≥ 3). Abbreviations: BB, hardwood BLN birch lignin; ep, ethanol precipitated; epBLN‐GGM, ethanol‐precipitated galactoglucomannan isolated using the BLN process; GGM, galactoglucomannan; GX, glucuronoxylan; LB, softwood kraft LignoBoost; LNPs, lignin nanoparticles; PB, wheat straw/Sarkanda grass alkali Protobind 1000; sd, spray dried.

**Figure 4:**
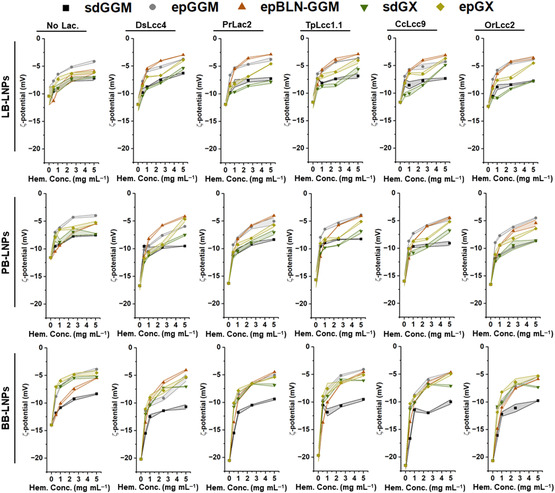
Stability of LNPs suspensions in 25 mm citric acid pH 3 by dynamic light scattering in terms of their surface charge, after incubation with the different laccases (1000 nKat g^−1^ of lignin) and hemicelluloses (0.5–5 mg mL^−1^), at pH 5 and RT for 24 h. Control samples were also analyzed in the absence of laccase and/or hemicelluloses. The lines between the symbols are to guide the eye, and their width represents the s.d. of each measured point (n ≥ 3). *Abbreviations:* BB, hardwood BLN birch lignin; ep, ethanol precipitated; epBLN‐GGM, ethanol‐precipitated galactoglucomannan isolated using the BLN process; GGM, galactoglucomannan; GX, glucuronoxylan; LB, softwood kraft LignoBoost; LNPs, lignin nanoparticles; PB, wheat straw/Sarkanda grass alkali Protobind 1000; sd, spray dried.

The stability of LNPs at wide range of pH is desired to increase the potential application of LNPs in different areas, including biomedical and food sciences. In our previous study, we evaluated the effect of pH (3–8) on the stability of LNPs, where we noticed that the bare LNPs start to experience aggregation at pH 3.^[^ ^33^ ^]^ Based on these observations, we assessed the effect of hemicellulose content (0.5–5 mg mL^−1^) to coat the LNPs on their stability, after incubating the resulting LNPs with citric acid at pH 3, in terms of average size (**Figure** **3**) and ζ‐potential (**Figure** **4**). As expected, the size of bare LNPs without any treatment was radically increased at acidic pH to over 600 nm (Figure 3), due to their aggregation when the pH gets closer to the isoelectric point and the carboxyl groups become protonated, inducing the intermolecular hydrogen bonding between particles.^[^ ^33^, ^41^ ^]^ Without any laccase treatment, the size of hemicellulose‐coated LB‐, PB‐, and BB‐LNPs substantially decreased from ≈600–1250 to 120–180 nm after their incubation at pH 3, suggesting that the physical adsorption of hemicelluloses occurred at the LNP surface. This adsorption took place even at the lowest concentration of hemicelluloses, which could be due to the noncovalent interactions between the abundant hydroxyl groups on both lignin and hemicelluloses. Similar nonionic interactions were observed between cellulose and hemicelluloses, and recent evidence has shown that hemicellulose adsorption can be entropically driven, with a substantial contribution of the release of structured water around the polymer and the hydrated cellulose surface.^[^ ^42^, ^43^, ^44^ ^]^

The treatment of LNPs with the fungal laccases resulted in an improved stability of the surface of LB‐, PB‐, and BB‐LNPs without any hemicellulose coating, shown by the decrease in their size compared to untreated control, in particular for the PB‐ and BB‐LNPs. As shown in our previous study, after laccase‐catalyzed modification of both PB‐ and BB‐LNPs, the amount of carbonyl groups increased as a consequence of the Cα‐oxidation, along with the decrease of phenolic content and formation of quinone after reaction.^[^ ^23^ ^]^ In fact, the Fourier‐transform infrared (FTIR) spectra of both PB‐ and BB‐LNPs after laccase treatment showed a simultaneous reduction of the band at 1720 cm^−1^ ascribed to the stretching of free carboxyl groups and the appearance of the peak at 1660 cm^−1^ corresponding to the conjugated C=O ketone (Figure S7, Supporting Information).^[^ ^45^ ^]^ Therefore, the protonation of free —COOH groups on the laccase‐treated LNP surface at acidic pH was reduced, and consequently, the LNP aggregation did not take place. Regarding the LB‐LNPs, the presence of free —COOH groups is higher than for PB‐ and BB‐LNPs as suggested by the more intense absorption peak at 1720 cm^−1^ (Figure S7, Supporting Information), leading to the protonation of the remaining —COOH groups at the LNP surface and consequent less extensive aggregation phenomenon compared to the untreated LB‐LNP control.

After adding the hemicelluloses and laccases on the LB‐LNPs, the average size of these LNPs incubated in acidic pH dramatically decreased according to the concentration of hemicellulose that was added (Figure 3). The density of the hemicellulose on the LNP surface seems to positively impact the LNP size at acidic conditions, that is, the higher the amount of hemicellulose for the reaction, the lower the LNP size. Furthermore, the epGX/GGMs‐coated LB‐LNPs exhibited better stability than the respective sdGX/GGM‐coated LNPs, whose average size decreased from 500–1450 nm to ≈140 nm (LB‐LNPs), probably due to the lower lignin content in their composition (see Table S2, Supporting Information) that can contribute to further increased stability of LNPs. Regarding the stability of PB‐ and BB‐LNPs at pH 3, their average size slightly decreased after hemicellulose coating, but the differences were not as pronounced as for the LB‐LNPs, because the untreated PB‐ and BB‐LNPs already exhibited great stability at acidic conditions.

The presence of different functional groups on the nanoparticle surface is closely related to the surface charge (ζ‐potential) and consequent stability of the nanoparticles; thus, tailoring their surface can improve their biological response and colloidal stability.^[^ ^39^ ^]^ Without any treatment, the ζ‐potential of the LNPs tends to increase as the pH gets highly acidic, as a consequence of the protonation of the carboxylic groups. However, the laccase‐treated LNPs incubated at acidic pH showed lower ζ‐potential than the untreated controls (Figure 4), in particular for the PB‐ and BB‐LNPs, decreasing from −11 to −17 mV (PB‐LNPs) or −14 to −23 mV (BB‐LNPs), due to the increase in the conjugated C=O groups after laccase‐induced oxidation. As expected, the ζ‐potential values increased even further after the functionalization of LNPs with the different hemicelluloses. Moreover, the epGX/GGMs‐coated LNPs presented higher ζ‐potential than the respective sdGX/GGM‐coated LNPs, due to the lower lignin content after ethanol precipitation of hemicelluloses that provided slightly more positive ζ‐potential values to the hemicelluloses (see Table S2, Supporting Information for the ζ‐potential values of hemicelluloses in MQ‐water). However, even when the surface charge gets close to neutral, the LNPs do not experience aggregation possibly due to the steric repulsion between LNPs given by the hemicellulose coating that prevents the LNP aggregation, suggesting an effective functionalization of the LNPs.

### 2.4 Chemical Characterization of Lignin–Hemicellulose Hybrid NPs Suspensions

Here, we aim to study which types of changes occurred on the LNP surface after reaction with hemicelluloses, with and without the laccase treatment. For this, we have set the hemicelluloses concentration in the reaction mixture at 2.5 mg mL^−1^, because their performance in terms of LNP size, surface charge, and stability at acid conditions was similar to the highest concentration tested (5 mg mL^−1^), and better than the lowest concentrations of hemicelluloses. During the laccase reaction to oxidize lignin and reduce O_2_ the removal of an electron and a proton from the phenolic hydroxyl groups occurs, to produce phenoxy radicals that can be stabilized by resonance in the aromatic structure of lignin.^[^ ^46^ ^]^ Perna et al. discovered that the laccase‐induced oxidation of birch lignin leads to the *in situ* production of micromolar levels of H_2_O_2_ in the reaction supernatant, possibly through a reaction between lignin phenoxy radicals and O_2_ after intramolecular transfer of a hydrogen atom from the Cα position, removal of hydroperoxyl radical to yield a quinone, and finally, the assimilation of a second hydrogen atom from lignin or reaction with another hydroperoxyl radical to form H_2_O_2_.^[^ ^47^ ^]^ In our previous study, we observed that the H_2_O_2_ levels in the supernatants were substantially higher for the hardwood BB‐LNPs than for both softwood LB‐LNPs and Sarkanda grass PB‐LNPs, in particular after 1 h of reaction.^[^ ^23^ ^]^ This phenomenon can be ascribed to the fact that BB‐LNPs contain a higher percentage of S units than the other two types of lignin, which preferably leads to the formation of Cα=O, which is in line with the results reported by Perna et al. Here, we quantified the H_2_O_2_ levels in the supernatants after reacting the LNPs with the different hemicelluloses (2.5 mg mL^−1^) for 1 h (Figure S8, Supporting Information). As expected, the laccase treatment of hardwood BB‐LNPs induced the highest production of H_2_O_2_, increasing from 3.8 μm (control) up to 10.2 μm after laccase incubation, whereas the softwood‐derived LB‐LNPs slightly increased from 0.46 (control) to 0.60 μm. Additionally, the incubation of LB‐LNPs with all hemicelluloses induced an increased production of H_2_O_2_. However, the reaction of laccase‐treated PB‐ and BB‐LNPs with sd/epGGMs reduced the H_2_O_2_ levels in the supernatant, probably due to the coating or “hindering” of the S units on the LNP surface by the softwood‐derived GGMs, which contain G units that are not as prone as the S units to the formation of Cα=O that ultimately leads to the production of H_2_O_2_. On the other hand, the incubation of all LNPs with hardwood‐derived GXs induced an augmented production of H_2_O_2_ levels up to about 17 μm, due to the higher content of S units in the GX composition (Table S2, Supporting Information). These observations confirmed the involvement of the S units in the structure of PB‐LNPs, BB‐LNPs, and GXs in the production of H_2_O_2_ after laccase oxidation, as a consequence of the preferable formation of Cα=O after the release of hydroperoxyl radical to ultimately form H_2_O_2_.

The changes in the phenolic hydroxyl content after laccase treatment of LNPs were also monitored in order to understand the mechanism of oxidation of the different LNPs and also the variation after hemicellulose coating of LNPs. For that, we measured the changes in the total phenolic content of the LNPs using the Folin–Ciocalteu method,^[^ ^33^ ^]^ after reacting the LNPs with hemicelluloses (2.5 mg mL^−1^) and laccases (1000 nKat g^−1^ of lignin) for 24 h (Figure S9, Supporting Information).

**Figure 5:**
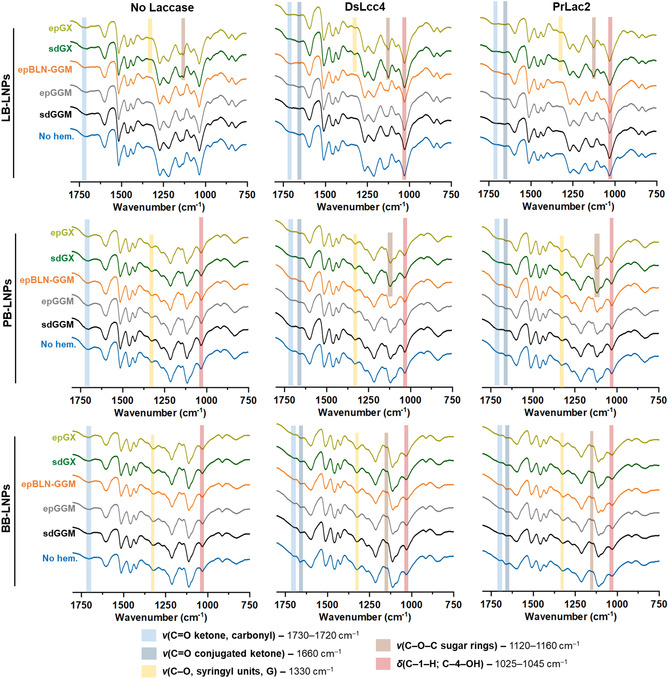
ATR‐FTIR spectra of LNPs, after their incubation with the different laccases (1000 nKat g^−1^ of lignin) and hemicelluloses (2.5 mg mL^−1^), at pH 5 and RT for 24 h. Control samples were also analyzed in the absence of laccase and/or hemicelluloses. Abbreviations: BB, hardwood BLN birch lignin; ep, ethanol precipitated; epBLN‐GGM, ethanol‐precipitated galactoglucomannan isolated using the BLN process; GGM, galactoglucomannan; GX, glucuronoxylan; LB, softwood kraft LignoBoost; LNPs, lignin nanoparticles; PB, wheat straw/Sarkanda grass alkali Protobind™ 1000; sd, spray dried.

The laccase‐induced oxidation of softwood LB‐LNPs, containing mainly G unit in their composition, preferably takes place in the side‐chains with the formation of dimeric products through the 5‐position of the aromatic ring that is free for crosslinking.^[^ ^23^ ^]^ Thus, changes in the OH groups of LB‐LNPs were not pronounced, as the Cα‐oxidation did not happen to the same extent as for BB‐LNPs, whose preferable oxidation mechanism was the Cα‐OH oxidation along with the decrease of phenolic hydroxyl content. Confirming these observations, and as shown in Figure S9, Supporting Information, the total phenolic content of both softwood LB‐LNPs did not decrease as much as for PB‐ and BB‐LNPs after laccase treatment, because both PB‐ and BB‐LNPs present higher percentage of S units that are more prone to experience an oxidation of Cα‐OH to form Cα=O, and consequently, the total hydroxyl content is reduced after laccase treatment. In addition, the coating of PB‐ and BB‐LNPs with sdGGM resulted in an augmented phenolic content than for LB‐LNPs after laccase treatments, due to the addition of G unit‐rich lignin moieties of sdGGM into the LNP surface, contributing to the increased OH content that did not experience Cα‐oxidation. Contrarily, the coating of LNPs with S units‐rich sdGX did not increase the total phenolic content of LNPs, because the Cα—OH in the S units experienced oxidation to form Cα=O, leading to the decrease in the OH content. Then, we analyzed and compared the FTIR spectra of the LNPs to assess the chemical modifications on the LNP surface after functionalization with hemicelluloses (**Figure** **5** and Figure S10, Supporting Information). The FTIR spectra showed the typical bands of lignin, such as unconjugated —COOH groups (1720–1730 cm^−1^), carbonyl groups at α‐ and γ‐locations (1600 cm^−1^), aromatic structures (1427–1512 cm^−1^), S units (1130, 1230, and 1330 cm^−1^), and G units (1150–1270 cm^−1^).^[^ ^10^, ^48^ ^]^ After laccase treatment of LNPs, there was a reduction of the band at ≈1720 cm^−1^ attributed to the stretching of carbonyl and carboxyl groups, and the appearance of the peak at 1660 cm^−1^ ascribed to the conjugated C=O ketone, which was more accentuated for the S units‐rich PB‐ and BB‐LNPs, as a result of the laccase oxidation mechanism. Moreover, after the addition of hemicelluloses, either by adsorption or after laccase treatments, an increased intensity of specific bands ascribed to the hemicelluloses could be seen at 1330 cm^−1^ (stretching vibrations of C—O bonds on the S units of GX), 1120–1260 cm^−1^ (C—O—C stretching in sugar rings), and also 1025–1045 cm^−1^ (C—1—H and C—4—OH bending vibrations in sugars).^[^ ^49^ ^]^

**Figure 6:**
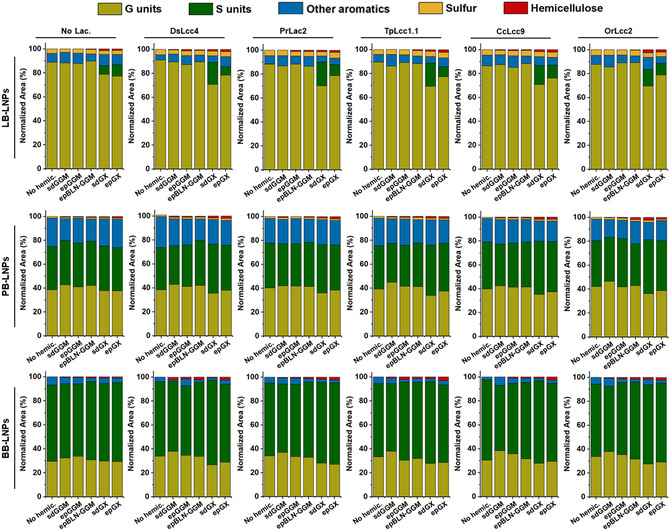
Py‐GCMS analysis of LNPs after their incubation with the different laccases (1000 nKat g^−1^ of lignin) and hemicelluloses (2.5 mg mL^−1^), at pH 5 and RT for 24 h. Control samples were also analyzed in the absence of laccase and/or hemicelluloses. Abbreviations: BB, hardwood BLN birch lignin; ep, ethanol precipitated; epBLN‐GGM, ethanol‐precipitated galactoglucomannan isolated using the BLN process; GGM, galactoglucomannan; GX, glucuronoxylan; LB, softwood kraft LignoBoost; LNPs, lignin nanoparticles; PB, wheat straw/Sarkanda grass alkali Protobind™ 1000; sd, spray dried.

The modifications on the LNP chemical composition after hemicellulose coating were also evaluated using Py‐GCMS, which allowed the analysis of LNPs by chromatographic separation and mass‐spectrometric identification of the compounds released after the pyrolytic breakdown of hemicellulose‐coated LNP samples, with and without laccase treatments. The peak areas were normalized for the total percentage of detected products, which were classified according to the type of lignin unit (i.e., G, S, and other aromatics), and the presence sulfur and hemicellulose derivatives (**Figure** **6**). As expected, the amount of lignin varies according to the source and processing conditions of the water‐soluble hemicellulose fraction (Table S2, Supporting Information): sdGGM (14.4%), epGGM (4.1%), epBLN‐GGM (1.3%), sdGX (18.3%), and epGX (12.9%). The lignin units’ composition in the hemicelluloses also differ according to the hemicellulose source: softwood GGM presented mainly G units, while hardwood GX exhibited mostly S units. In addition, all hemicelluloses presented a high percentage of acetic acid (Figure S11, Supporting Information), which is in accordance with previous studies that detected high levels of acetic acid, as a result of the elimination of *O*‐acetyl groups in the hemicelluloses structure.^[^ ^50^, ^51^ ^]^ Due to the intrinsic higher acetylation degree, the percentage of acetic acid was increased in GXs samples compared to the GGMs samples.^[^ ^31^ ^]^

As expected, acetic acid was the main carbohydrate‐derived compound released after the pyrolytic breakdown of hemicellulose‐coated LNPs. Generally, the percentage of hemicellulose‐derived compounds released after the pyrolytic breakdown of hemicellulose‐coated LNPs increased to a maximum of 1.2% for the hemicellulose adsorbed onto the LNPs and 2.5% for the lignin–hemicellulose hybrid NPs assisted by laccases. For the three types of LNPs, the variation of the lignin‐derived compounds released after the pyrolytic breakdown LNPs coated with ep‐hemicelluloses was not as noticeable as for the sd‐hemicelluloses due to the lower lignin content after ethanol precipitation treatment of the spray‐dried hemicelluloses.

Regarding the LB‐LNPs (Table S3, Supporting Information), the percentage of G units‐derived compounds after functionalization with GGMs did not change significantly, as the LB‐LNPs controls presented already high percentage of G‐type compounds (> 86%). On the other hand, the percentage of S units‐derived compounds after coating the LNPs with S units‐rich GX experienced a pronounced increase, along with the decrease of the percentage of G‐type compounds. Similar to the carbohydrate‐derived compounds, the sdGX‐functionalized LB‐LNPs treated with laccases exhibited at least double the percentage of S‐type compounds than the nontreated laccase samples, which can suggest a laccase‐induced linkage between the LNPs and sdGX happening through the lignin moieties in the GX. As for the hemicellulose‐functionalized PB‐LNPs (Table S4, Supporting Information), which present a similar proportion of G and S units in their composition (≈37% of each), the percentage of G‐type compounds released after the pyrolytic breakdown of LNPs increased up to 4% when the LNPs were coated with sdGGM. In addition, the percentage of S‐type compounds increased by 5% after functionalization of the LNPs with sdGX after treatment with laccases, along with the reduction in the total percentage of G‐type compounds. These differences were even more pronounced in the hemicellulose‐functionalized BB‐LNPs (Supporting Information Table S5), which exhibit in their composition a higher percentage of S units (≈64%) compared to the PB‐LNPs. When the sdGGM was adsorbed into the BB‐LNPs, the percentage of G‐type compounds increased by ≈2%, while the laccase treatment of these samples led to an increase of 4%–8%, suggesting the reaction of LNPs with the G units present in the sdGGM. In a similar way, the percentage of S‐units derived compounds increased up to 8% after coating the BB‐LNPs with sdGX assisted by laccases, accompanied by the reduction in the percentage of G‐type compounds. Overall, the percentage of carbohydrate‐derivative compounds given by the acetic acid percentage after pyrolytic breakdown of the hemicellulose adsorbed on the LNPs, that is, without any laccase treatment, was lower than that of the hemicellulose‐coated LNPs assisted by laccases. Along with the fact that higher percentage of lignin units are present in the composition of LNPs coated with sd‐hemicelluloses after treatment with laccases, our results suggest that the laccases can play an active role in the linkage between the lignin moieties in the hemicelluloses and the LNP surface.

**Figure 7:**
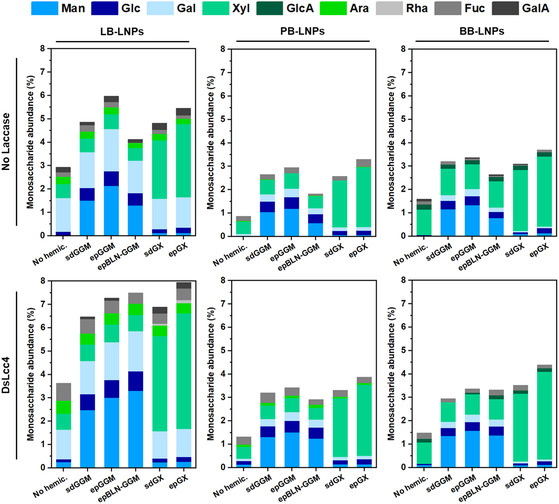
Monosaccharide composition of LNPs functionalized with hemicelluloses (2.5 mg mL^−1^) by spontaneous absorption (no laccase), and treated with DsLcc4 (1000 nKat g^−1^ of lignin), at pH 5 and RT for 24 h, determined after acid methanolysis followed by gas chromatography‐flame ionization detection (GC‐FID). Control samples were also analyzed in the absence of hemicelluloses. Abbreviations: BB, hardwood BLN birch lignin; ep, ethanol precipitated; epBLN‐GGM, ethanol‐precipitated galactoglucomannan isolated using the BLN process; GGM, galactoglucomannan; GX, glucuronoxylan; LB, softwood kraft LignoBoost; LNPs, lignin nanoparticles; PB, wheat straw/Sarkanda grass alkali Protobind™ 1000; sd, spray dried.

The analysis of the carbohydrate composition in the LNPs was carried out in order to evaluate the changes after functionalization of LNPs with hemicelluloses (**Figure** **7**, Table S6, Supporting Information). First, we assessed the monosaccharide distribution in the hemicelluloses used for the reactions (Table S2, Supporting Information). As expected, softwood‐derived sd‐, ep‐, and epBLN‐GGM exhibited mannose as their major constituent (50%–64%), followed by small fractions of glucose (12.3%–17.1%), galactose (5.9%–12.2%), and xylose (5.8%–11.0%). GGMs typically contain β‐d‐mannopyranosyl units and β‐d‐glucopyranosyl units randomly arranged and linked by 1 → 4 bonds, which present α‐d‐galactopyranosyl units linked through 1 → 6 bonds. The presence of xylose in softwood GGMs is due to the existence of arabinoglucuronoxylans.^[^ ^52^ ^]^ On the other hand, hardwood‐derived sd‐ and ep‐GX exhibited mainly xylose in their composition (>91%), derived from glucuronoxylans. Due to the difficulty in separating the lignin from hemicellulose, lignins present some carbohydrate moieties in their composition, which are covalently bound to form LCCs.^[^ ^52^, ^53^ ^]^ Therefore, we evaluated the monosaccharide composition of the technical lignin and respective LNPs (Figure S1b and Table S1, Supporting Information), and the differences observed in the proportion of monosaccharides can be attributed to the type of LCC structures present in the lignin composition, which vary according to the lignin/hemicellulose species.^[^ ^53^ ^]^ Usually, the composition of LCCs extracted from softwoods involves the crosslinking of lignin with galactoglucomannans and xylans,^[^ ^53^, ^54^ ^]^ which can explain the higher content of galactose and xylose in the LB lignin/LNPs structure (Figure S1b, Supporting Information). In hardwoods, the LCC structure implies the linkage of lignin and glucuronoxylan by benzylether bonds, for example,^[^ ^53^, ^55^ ^]^ and therefore, the xylose was the main monosaccharide found in the BB lignin/LNPs composition. In nonwood biomass, such as grass, arabinoxylans play an important role in LCC linkage formation with lignin moieties,^[^ ^53^, ^56^ ^]^ which is consistent with our results showing that xylose was the main monosaccharide in PB lignins/LNPs composition, followed by arabinose. Finally, we assessed the changes in the monosaccharide composition of LNPs functionalized with hemicelluloses by spontaneous absorption (no laccase) or treated with *Ds*Lcc4 as representative of all the laccases (Figure 7, Table S6, Supporting Information). Generally, the percentage of carbohydrates in the LNP samples increased after functionalization with hemicelluloses, compared to the control samples that were submitted to the same reaction conditions, but without the hemicelluloses: for LB‐LNPs, the percentage of monosaccharides increased 1.4–2.2 times, while the PB‐ and BB‐LNPs exhibited 2.1–3.8 and 1.7–3.0 times more carbohydrates, respectively. In terms of carbohydrate composition, the coating of LNPs with the softwood‐derived sd‐, ep‐ or epBLN‐GGMs led to an obvious increase in the percentage of mannose, which is the major component of GGMs. The percentage of mannose in the LB‐LNPs increased to 1.3%–2.1% after adsorption of GGMs, and it was amplified after treatment with *Ds*Lcc4 (2.5%–3.3%). A similar trend was observed for both PB‐ and BB‐LNPs, before and after laccase treatment, respectively: 0.6–1.2 vs 1.3%–1.5% for PB‐LNPs and 0.8–1.3 vs 1.4%–1.6% for BB‐LNPs. The presence of glucose and galactose was also enlarged after functionalization of LNPs with GGMs. On the other hand, the LNPs coated with hardwood‐derived sd‐ and ep‐GXs exhibited an increased percentage of xylose in their composition, because of the glucuronoxylans present in the GXs structure. In addition, the percentage of xylose in GXs‐coated LB‐LNPs increased 4.2–5.3 times after GX adsorption, while the *Ds*Lcc4‐treated LB‐LNPs exhibited 6.0–7.3 times more xylose. Furthermore, the same tendency was noticed for both PB‐ and BB‐LNPs, before and after laccase treatment, respectively: 3.8–4.8 vs 4.9–6.0 times for PB‐LNPs and 2.4–2.7 vs 3.1–4.1 times higher percentage of xylose for BB‐LNPs. Overall, the increase in mannose (GGMs) and xylose (GXs) after laccase treatment of hemicellulose‐coated LNPs confirmed the results obtained with pyrolysis GCMS, where the laccase‐treated LNPs functionalized with hemicelluloses exhibited higher percentage of hemicellulose‐derived compounds, and the LNPs coated with sd‐GGM/GX presented higher percentage of lignin units in their composition.

**Figure 8:**
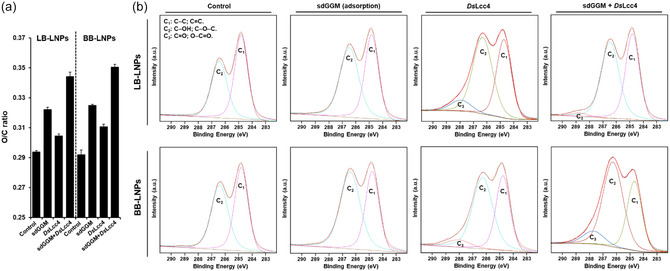
Oxygen/carbon (O/C) ratio a) and high‐resolution XPS C 1s spectra b) of LB‐ and BB‐LNP functionalized with sdGGM (2.5 mg mL^−1^) by spontaneous absorption (no laccase), and treated with DsLcc4 (1000 nKat g^−1^ of lignin), at pH 5 and RT for 24 h. Control samples were also analyzed in the absence of laccase and/or hemicellulose. Error bars represent the s.d. of each measured point (*n* = 3). Abbreviations: BB, hardwood BLN birch lignin; GGM, galactoglucomannan; LB, softwood kraft LignoBoost; LNPs, lignin nanoparticles; sd, spray dried.

Based on the previous observations obtained from Py‐GCMS and monosaccharide composition, we selected softwood LB‐ and hardwood BB‐LNPs coated with sdGGM after spontaneous adsorption and laccase‐assisted reaction (*Ds*Lcc4) in order to evaluate the changes in their surface chemical structure and composition, using X‐ray photoelectron spectroscopy (XPS). The wide‐scan spectra of LNP samples, along with the high‐resolution XPS spectra of O 1s, N 1s, and S 2p core levels, are represented in Figure S12, Supporting Information. Regarding the elemental composition calculated from the spectral survey (Table S7, Supporting Information), all the LB‐LNP samples presented a small amount of sulfur (0.54%–0.79%) as result of the isolation process, unlike the BB‐LNPs that do not contain any sulfur. Additionally, the LNPs before and after spontaneous adsorption of sdGGM presented traces of nitrogen (0.05%–0.18%), which increased to 0.21%–0.27% and 0.37%–0.52% after *Ds*Lcc4 treatment of LB‐LNPs and BB‐LNPs, respectively. This slight increase in the nitrogen content might be due to some adsorption of laccases on the LNP surfaces by electrostatic and hydrophobic interactions, which were not removed during washing step after reactions.^[^ ^57^, ^58^ ^]^ The oxygen to carbon (O/C) ratios increased after coating the LNPs with sdGGM (**Figure** **8**a), due to the higher oxygen content of hemicelluloses compared to the lignin core, as a result of the multiple hydroxyl (—OH) and ether (C—O—C) groups in the sugar units.^[^ ^59^ ^]^ Moreover, the absolute increase of the O/C ratio in the LNPs coated with sdGGM and treated with laccase is higher than that in the case of spontaneous adsorption of sdGGM, which is in line with the higher percentage of monosaccharides after laccase treatment. Finally, the deconvoluted C 1s spectra (Figure 8b) of the control samples and LNPs coated with sdGGM after spontaneous adsorption revealed two of signature peaks corresponding to the binding energies of C—C/C=C (284.4 eV) and C—O linkages in ethers and alcohols (286.4 eV). Furthermore, the intensity of the peak ascribed to the C—O linkages in ethers and alcohols at 286.4 eV in the sdGGM‐functionalized LNPs is higher than the intensity of C—C/C=C linkages at 284.4 eV, due to the addition of hemicellulose, which were even higher in the presence of laccase. Additionally, we observed the appearance of a third peak centered at ≈288.8 eV after treatment of LNPs with DsLcc4, ascribed to the presence of C=O bonds in acetals and esters,^[^ ^60^, ^61^ ^]^ as a result of the oxidation of LNPs. Altogether, these results indicate that the laccase treatment can incite a more efficient linkage between the LNP surface and the lignin moieties in hemicelluloses.

## 3 Conclusion

Designing and tailoring the nanoparticle surface allows the control of the functionality and application of the nanoplatforms, as it can have an impact in their dissolution, fate, and accumulation, as well as potential risks for human health and the environment. In this work, we developed a hybrid nanoplatform composed of two of the most abundant and renewable polymers on Earth, where the LNPs were successfully decorated with hemicelluloses in order to improve the colloidal stability at acidic pH of the resulting hybrid NPs. Then, we analyzed the effect of the lignin content in the hemicelluloses’ composition on the efficacy of LNP coating. As the concentration of hemicellulose in the reaction increased, the resulting hemicellulose‐coated LNPs showed improved stability at acidic pH, compared to the bare LNPs, due to the steric repulsion between LNPs provided by the hemicellulose coating. However, when the amount of hemicellulose for the reaction was set constant (2.5 mg mL^−1^), we observed that the chemical composition after pyrolytic breakdown of the resulting hybrid hemicellulose–LNPs exhibited a higher content of hemicellulose‐ and lignin‐derived compounds when the LNPs were treated with laccases, compared to the uncoated and spontaneously adsorbed hemicellulose–LNPs. In addition, monosaccharide composition changed after coating the LNPs with hemicellulose, and the percentage of carbohydrates was higher after laccase treatment of hemicellulose‐coated LNPs. Overall, our results suggested that the fungal laccases can play a role to produce an efficient linkage between the LNP surface and lignin‐richer hemicelluloses (i.e., sd‐GGM and ‐GX). Due to their increased stability at acidic pH, we envisioned to tailor the LNPs with sdGGM or sdGX to validate the stability and functional performance of the hemicellulose‐lignin hybrid nanoparticles as carriers for oral delivery of pharmaceuticals/nutraceuticals. We specifically propose to encapsulate hydrophobic molecules and study their release from the hybrid nanoparticles at acidic and physiological conditions, as well as evaluate the biocompatibility and mucoadhesive properties of the hybrid nanoparticles to permeate through the intestinal mucosal layer and facilitate the *trans*‐epithelial transport.

## 4 Experimental Section

4.1

4.1.1

##### Materials

Softwood kraft Lignoboost (LB) was provided by Stora Enso (Finland). Hardwood birch lignin (BB, Betula L.) was isolated using the BLN process, and obtained from CH‐Bioforce Oy (Finland). Protobind 1000 (PB) was extracted from wheat straw by the soda process and acquired from GreenValue SA (Switzerland). sd spruce galactoglucomannan (GGM) and birch glucuronoxylans (GX) were obtained from Luke (Finland). Both hemicelluloses were submitted to an ethanol precipitation (ep) approach to reduce their lignin content.^[^ ^32^, ^62^ ^]^ Ethanol‐precipitated galactoglucomannan isolated using the BLN process (epBLN‐GGM) was provided by CH‐Bioforce Oy (Finland). Acetone for high‐performance liquid chromatography (≥ 99.9 %), citric acid monohydrate, hydrochloric acid, anhydrous methanol, methanol, trimethylchlorosilane (TMSCI), bis(trimethylsilyl)trifluoroacetamide (BSTFA), heptane, galactose, fucose, and glucuronic acid were acquired from Sigma‐Aldrich (Finland). Glucose, xylose, mannose, and arabinose were acquired from Merck (Finland), while rhamnose and galacturonic acid were purchased from Fluka (Fisher scientific, Finland).

##### Heterologous Expression of Fungal Laccases

The heterologous expression of fungal laccases (*Ds*Lcc4, *Pr*Lac2, *Tp*Lcc1.1, *Cc*Lcc9, *Or*Lcc2) was done as detailed by Figueiredo et al.^[^ ^23^ ^]^ with some modifications. After the linearized plasmid constructs were transformed into *Pichia pastoris* X‐33 competent cells, the best laccase‐producing transformants were chosen using 2,2′‐azinobis(3‐ethylbenzathiazoline‐6‐sulfonate) (ABTS)‐plate assay. The selected transformants were first cultivated in 6 mL of yeast extract peptone dextrose [YPD; 1% (wt/v) yeast extract (Labema, Finland), 2% (wt/v) peptone (Labema, Finland), 2% (wt/v) D‐glucose] liquid medium at 30 °C with shaking (250 rpm) until OD_600_ was ≈7–8. Then, 6 × 400 mL of buffered glycerol‐complex medium [1% (wt/vol) yeast extract (Labema, Finland), 2% (wt/v) peptone (Labema, Finland), 100 mM potassium phosphate, pH 6.0, 1.34% (wt/v) YNB, 4 × 10%‐5% (wt/v) biotin, 1% (vol/vol) glycerol] was inoculated with ≈800 μL of transformant‐YPD cultivation, which were let to grow at 30 °C with shaking (250 rpm) for ≈17 h. The cells were pelleted by centrifugation at 22 °C, 4000 *g* for 5 min, and the obtained pellets were washed with phosphate‐buffered saline (137 mm NaCl, 2.7 mm KCl, 10 mm Na_2_PO_4_, 1.8 mm KH_2_PO_4_). The cell pellets were resuspended in buffered minimal medium (100 mm potassium phosphate, pH 6.0, 1.34% yeast nitrogen base (YNB), 4 × 10^−5^% biotin) supplemented with 0.3 mm CuSO_4_% and 3% v/v methanol. Laccase expression was controlled daily by 3% v/v methanol addition, and the induction was continued for 72 h at 20 °C. The extracellular laccase activity was followed daily by 2,6‐dimethoxyphenol (2,6‐DMP) as a substrate. The cultivation supernatant was collected by centrifugation (6000 *g*, 15 min, 4 °C). The cultivation medium was filtrated using 22 μm pore size polyethersulfone (PES)‐membrane (Millipore Express) and concentrated using Amicon‐pressurized ultrafiltration unit (Millipore; Stirred Cell 400 mL) and with Spin‐X UF 20 mL concentrator (Corning) with 5 kDa cutoff till the final volume of 1.5–2 mL.

##### Preparation of LNPs

The LNPs were prepared using the acetone nanoprecipitation approach that was previously reported by Farooq et al.^[^ ^63^ ^]^ and adapted by Figueiredo et al.^[^ ^33^ ^]^ Briefly, 2 g of technical lignins were dissolved in 200 mL of acetone/water 3:1 v/v mixture and stirred overnight, followed by filtration of the lignin solution using a glass microfiber filter (Whatman GF/F, pore size 0.7 μm). The obtained solution was rapidly poured into 400 mL of MilliQ‐water under vigorous stirring for 1 h. Acetone was further removed by evaporation under reduced pressure at 40 °C to obtain the LNPs dispersions. Finally, the LNP suspensions were centrifuged for 15 min at 50,000* g* and redispersed with MilliQ‐water using ultrasonication (Branson digital sonicator) at a frequency of 20 kHz, with 30% oscillation amplitude (100 W) for 60 s. LB, PB, and BB technical lignins were used as starting polymers to yield LB‐LNPs, PB‐LNPs, and BB‐LNPs, respectively.

##### Preparation of the Lignin–Hemicellulose Hybrid NPs by Spontaneous Adsorption and Laccase‐Assisted Reaction of Hemicelluloses with LNPs

All hemicelluloses (sdGGM, epGGM, epBLN‐GGM, sdGX, and epGX) were previously dissolved in 25 mm citric acid pH 5, at concentrations of 1, 2, 5, and 10 mg mL^−1^, overnight, and further centrifuged for 10 min at 20000* g* to remove the undissolved hemicelluloses. For both spontaneous adsorption and laccase‐assisted reactions, aqueous dispersions of LNPs in 25 mm citric acid pH 5 at final concentration of 1 mg mL^−1^ were mixed with the previously dissolved hemicelluloses solutions (diluted in the reaction to 0.5, 1, 2.5, and 5 mg mL^−1^). Finally, in the case of the laccase‐assisted reactions, all the laccases (*Ds*Lcc4, *Pr*Lac2, *Tp*Lcc1.1, *Cc*Lcc9, *Or*Lcc2) were added to the previous mixture at a dosage of 1000 nKat per gram (nKat g^−1^) of lignin and allowed to react for 24 h. Control samples were prepared in the same way, without any hemicellulose and/or laccase treatment. After reactions, the samples were centrifuged at 12500* g* for 15 min, washed twice with MilliQ‐water, and redispersed with MilliQ‐water for further analysis.

##### Dynamic Light Scattering

The average hydrodynamic diameter, PDI, and ζ‐potential of LNPs was measured by DLS, using a Malvern Zetasizer Nano ZS instrument (Malvern Instruments Ltd, UK). For that, the samples were diluted in MilliQ‐water at a concentration of 0.5 mg mL^−1^. Different time points (Day 1 and Week 7) were considered in order to evaluate the long term‐stability of the prepared LNPs.

##### Transmission Electron Microscopy

To confirm the size distribution and morphology of LNPs, the particles were visualized by TEM (Jeol JEM‐1400, Jeol Ltd., Tokyo, Japan), using an acceleration voltage of 80 kV. For the sample preparation, a droplet of LNPs’ dispersion was placed on a carbon‐coated copper grid, blotted using a filter paper, and then air dried before analysis.

##### Pyrolysis Gas Chromatography Mass Spectrometry (Py‐GCMS)

Pyrolysis was performed with an EGA/PY3030D Multishot pyrolyzer (Frontier Laboratories, New Ulm, MN, USA) equipped with an AS‐1020 E Autoshot autosampler. The pyrolyzer was coupled to GC‐MS using a Trace GC equipped with a DB‐1701 fused‐silica capillary column (30 m × 0.25 mm i.d. 0.25 μm film thickness) coupled to a DSQ‐II mass spectrometer (Thermo Scientific, Waltham, MA, USA). Samples were weighed using a XP6 excellence‐plus microbalance (Mettler Toledo, Columbus, OH, USA). Pyrolysis of LB‐, PB‐, and BB‐LNPs (100−120 μg) was carried out at 500 °C for 1 min with an interface temperature of 320 °C. Pyrolysis products were injected on the column via split injection (at 300 °C) with a split ratio of 25, and helium was used as carrier gas with constant flow at 1.5 mL min^−1^. The GC oven was programmed from 70 °C (2 min) to 270 °C at 5 °C min^−1^ and held at 270 °C for 15 min. MS etection was used with EI at 70 eV, a source temperature of 250 °C, a scan range of *m/z* 45−450, and a scan rate of 4.0 scans s^−1^. Compounds were identified by comparing retention time and mass spectrum with standards, the NIST library.

##### Phenolic Content

The total amount of phenolic hydroxyl groups was determined using a spectrophotometric method based on the Folin–Ciocalteu reagent.^[^ ^33^, ^64^ ^]^ For that, 25 μL of aqueous dispersions of LNP (0.5 mg mL^−1^) were diluted with 900 μL of MilliQ‐water and further mixed with 75 μL Folin–Ciocalteu reagent. After ≈6 min, 250 μL of sodium carbonate solution (20 w/w%) was added, and the resulting mixtures were mixed and kept at 40 °C for 30 min. Finally, the absorbance at 760 nm of the blue‐colored samples was measured using a Varioskan Flash plate reader (Thermo Fisher Scientific, Inc., NY, USA). The amount of free phenolic groups was quantified from standard curves based on vanillin (4‐hydroxy‐3‐methoxybenzaldehyde).

##### Measurement of H_*2*_ *O*_*2*_

The Amplex Red reagent (10‐acetyl‐3,7‐dihydroxyphenoxazine), in combination with horseradish peroxidase (HRP), was used to measure the generation of hydrogen peroxide (H_2_O_2_). After incubation with the laccases and/or hemicelluloses, the LNP suspensions were centrifuged and 50 μL of the supernatant was collected and placed in a 96‐well plate, and 50 μL of Amplex Red reagent was added and reacted for 30 min. In the presence of peroxidase, the Amplex Red reagent reacts with H_2_O_2_ to produce the red‐fluorescent oxidation product resorufin (*λ*_ex_ = 571 nm and *λ*_em_ = 585 nm) that was quantified with Varioskan Flash plate reader (Thermo Fisher Scientific Inc., USA).

##### Nuclear Magnetic Resonance Spectroscopy

For the ^31^P NMR spectroscopy, 30 mg of each sample was dissolved in 400 μL of a 1:1.6 mixture of CDCl_3_/pyridine after which 100 μL of an internal standard solution (e‐HNDI, *endo‐N‐*hydroxy‐5‐norbornene‐2,3‐dicarboximide, 0.12 m in CDCl3/pyridine 1:1.6) was added, followed by 50 μL of a relaxation agent [Cr(acac)3, 11.4 mg mL^−1^ in CDCl_3_/pyridine 1:1.6]. Finally, 100 μL of a phosphitylation reagent Cl‐TMDP (2‐chloro‐4,4,5,5‐tetramethyl‐1,3,2‐dioxaphospholane) was added and the solution was stirred for 1 h to ensure complete derivatization before transferring the samples to NMR tubes. The spectra were acquired on a Bruker AVANCE III spectrometer operating at 500.10 MHz (^1^H) and 202.44 MHz (^31^P), equipped with a BB/1H SmartProbe. The data was acquired using a standard pulse program for quantitative ^31^P NMR spectroscopy, using inverse‐gated decoupling (zgig). The relaxation delay was set to 10 s, and the acquisition time to 3.3 s resulting in a total repetition time of 13.3 s to ensure complete relaxation between scans. A total of 64 scans were acquired on each sample. Spectra were recorded from 117 to 166 ppm to place the region of interest (≈130–154 ppm) in the center of the spectrum.

##### Lignin Nanoparticles (LNP) Dispersion Stability

The colloidal stability of LNP suspensions at 0.5 mg mL^−1^ (2 mL) during storage in 4 mL vials was monitored using Turbiscan Lab Expert (Formulaction, Toulouse, France) at the wavelength of 800 nm (near‐infrared light). The transmitted light intensity through the LNP suspensions (height range of 19–29 mm) was measured using Turbisoft version 1.2 (Formulaction, Toulouse, France) software. Results were presented in terms of percentage of ΔTransmission, calculated by the difference of the transmitted light at the predefined time point and the initial transmitted light through the LNP suspensions. The measurements were performed just after LNP preparation and up to 30 days of storage.

##### Stability of Lignin–Hemicellulose Hybrid NPs at pH 3

After reacting the LNPs with hemicelluloses, the stability of the lignin‐hemicellulose hybrid NPs was evaluated by incubating the LNPs at concentration of 0.5 mg mL^−1^ in 25 mm citric acid, pH 3. Afterward, the average hydrodynamic diameter and ζ‐potential of the LNP suspensions were measured by DLS using the Malvern Zetasizer Nano ZS instrument.

##### Fourier‐Transform Infrared Spectroscopy

The FTIR spectra of the technical lignins and LNPs were recorded using SpectrumOne (PerkinElmer, Turku, Finland), equipped with a universal attenuated total reflectance accessory. The FTIR spectra were recorded at room temperature between 4000 and 600 cm^−1^ with a resolution of 4 cm^−1^ and number of 64 scans, and the baseline was corrected using the built‐in software.

##### Acid Methanolysis

Monosaccharide composition was determined by gas chromatography‐flame ionization detection (GC‐FID, HP 6890 N, Agilent Technologies, Waldbronn, Germany) of silylated methanolyzed monosaccharide derivatives.^[^ ^31^, ^65^ ^]^ About 10 mg of hemicellulose samples were weighed in a pear‐shaped flask and suspended in 2 mL of 2 m hydrochloric acid in anhydrous methanol. The sample was incubated in an oven at 100 °C for 3 h. After cooling to room temperature 100 μL pyridine were added for neutralization and the suspension was diluted to 10 mL with methanol. An aliquot of 600 μL methanolized products were transferred to glass tubes, 100 μL methanol containing 1 mg mL^−1^ sorbitol (internal standard for neutral and acid monosaccharides determination) was added, and the sample was dried at 50 °C under a nitrogen flow. Next, 100 μL of pyridine and 100 μL of TMSCI/BSTFA 1:99 v/v were added to dry samples and silylation was performed at room temperature overnight. Silylated products were dried at 50 °C under a nitrogen flow, dissolved in 1 mL heptane, filtered through syringe filter (acrodisc, 0.45 μm) to GC vials, and analyzed by GC‐FID equipped with a DB‐1 column (30 m, i.d. 0.25 mm, 0.25 μm film). For improving the monosaccharide quantification in lignin samples, acid methanolysis and silylation methods were optimized as follows: about 20 mg of freeze‐dried lignin sample (adjusted to contain about 3 mg hemicelluloses) were used in acid methanolysis. After incubation and neutralization no dilution was performed after the reaction (i.e., the 2 mL methanolized products were subjected to silylation). In silylation, the volume of 200 μL TMSCI/BSTFA 1:99 v/v was added to dry lignin samples instead of the 100 μL added to hemicellulose samples to ensure complete derivatization of monosaccharides. For monosaccharide determination of hemicellulose and lignin samples, 1 mL of sample was injected and eluted at 20:1 split ratio. The temperature program consisted of keeping the injected sample at 150 °C for 3 min, then increasing from 2 °C min^−1^ to 186 °C, 1 °C min^−1^ to 200 °C, 20 °C min^−1^ to 300 °C, and holding at this temperature for 1 min. Calibration curve (with internal standard calibration) was prepared using glucose, xylose, mannose, galactose, arabinose, rhamnose, fucose, glucuronic acid, and galacturonic acid and using the highest peak of each corresponding monosaccharide. Monosaccharide determination was performed with at least two parallel replicates and the results, reported as anhydrous monosaccharide, were subjected to one‐way analysis of variance and Tukey test for pairwise comparison of means at 5% significance level using the Origin 2022b software.

##### X‐Ray Photoelectron Spectroscopy (XPS) Analysis

The measurements were performed with a Kratos AXIS Ultra DLD X‐ray photoelectron spectrometer using a monochromated Al Kα X‐ray source (1486.7 eV) run at 100 W. A pass energy of 80 eV and a step size of 1.0 eV were used for the survey spectra, while a pass energy of 20 eV and a step size of 0.1 eV were used for the high‐resolution spectra. Photoelectrons were collected at a 90° take‐off angle under ultrahigh‐vacuum conditions, with a base pressure typically below 1 × 10^−9^ Torr. The diameter of the beam spot from the X‐ray was 1 mm, and the area of analysis for these measurements was 300 × 700 μm. Both survey and high‐resolution spectra were collected from three different spots on each sample surface to check for homogeneity and surface charge effects. All spectra were charge corrected to the position of C—C carbon at 284.8 eV.

## Conflict of Interest

P.F., D.M.C., M.H.L., K.S.H., and K.S.M. have a related patent submitted, entitled “Method for producing lignin‐hemicellulose hybrid nanoparticles or lignin nanoparticles” (no. PCT/FI2024/050120).

## Supporting information

Supplementary Material

## Data Availability

The data that support the findings of this study are available in the supplementary material of this article.

## References

[1] M. Graglia , N. Kanna , D. Esposito , ChemBioEng. Rev. 2015, 2, 377.

[2] A. J. Ragauskas , G. T. Beckham , M. J. Biddy , R. Chandra , F. Chen , M. F. Davis , B. H. Davison , R. A. Dixon , P. Gilna , M. Keller , P. Langan , A. K. Naskar , J. N. Saddler , T. J. Tschaplinski , G. A. Tuskan , C. E. Wyman , Science 2014, 344, 1246843. 24833396 10.1126/science.1246843

[3] S. Constant , H. L. J. Wienk , A. E. Frissen , P. De Peinder , R. Boelens , D. S. Van Es , R. J. H. Grisel , B. M. Weckhuysen , W. J. J. Huijgen , R. J. A. Gosselink , P. C. A. Bruijnincx , Green Chem. 2016, 18, 2651.

[4] P. Figueiredo , K. Lintinen , J. T. Hirvonen , M. A. Kostiainen , H. A. Santos , Prog. Mater Sci. 2018, 93, 233.

[5] M. N. Collins , M. Nechifor , F. Tanasă , M. Zănoagă , A. McLoughlin , M. A. Stróżyk , M. Culebras , C. A. Teacă , Int. J. Biol. Macromol. 2019, 131, 828. 30872049 10.1016/j.ijbiomac.2019.03.069

[6] W. D. H. Schneider , A. J. P. Dillon , M. Camassola , Biotechnol. Adv. 2021, 47, 107685. 33383155 10.1016/j.biotechadv.2020.107685

[7] A. Duval , M. Lawoko , React. Funct. Polym. 2014, 85, 78.

[8] M. H. Sipponen , H. Lange , C. Crestini , A. Henn , M. Österberg , ChemSusChem 2019, 12, 2039. 30933420 10.1002/cssc.201900480 PMC6593669

[9] M. Österberg , M. H. Sipponen , B. D. Mattos , O. J. Rojas , Green Chem. 2020, 22, 2712.

[10] P. Figueiredo , C. Ferro , M. Kemell , Z. Liu , A. Kiriazis , K. Lintinen , H. F. Florindo , J. Yli‐Kauhaluoma , J. Hirvonen , M. A. Kostiainen , H. A. Santos , Nanomedicine 2017, 12, 2581. 28960138 10.2217/nnm-2017-0219

[11] J. P. Martins , P. Figueiredo , S. Wang , E. Espo , E. Celi , B. Martins , M. Kemell , K. Moslova , E. Mäkilä , J. Salonen , M. A. Kostiainen , C. Celia , V. Cerullo , T. Viitala , B. Sarmento , J. Hirvonen , H. A. Santos , Bioact. Mater. 2022, 9, 299. 34820572 10.1016/j.bioactmat.2021.08.007 PMC8586719

[12] P. Figueiredo , A. Lepland , P. Scodeller , F. Fontana , G. Torrieri , M. Tiboni , M. A. Shahbazi , L. Casettari , M. A. Kostiainen , J. Hirvonen , T. Teesalu , H. A. Santos , Acta Biomater. 2021, 133, 231. 33011297 10.1016/j.actbio.2020.09.038

[13] P. Figueiredo , M. H. Sipponen , K. Lintinen , A. Correia , A. Kiriazis , J. Yli‐Kauhaluoma , M. Österberg , A. George , J. Hirvonen , M. A. Kostiainen , H. A. Santos , Small 2019, 15, 1901427. 10.1002/smll.201901427 PMC8042775 31062448

[14] A. Bjelić , M. Grilc , U. Novak , B. Hočevar , B. Likozar , Rev. Chem. Eng. 2022, 38, 243.

[15] M. B. Agustin , D. M. de Carvalho , M. H. Lahtinen , K. Hilden , T. Lundell , K. S. Mikkonen , ChemSusChem 2021, 14, 4615. 34399033 10.1002/cssc.202101169 PMC8597079

[16] Y. Zheng , A. Moreno , Y. Zhang , M. H. Sipponen , L. Dai , Trends Chem. 2024, 6, 62.

[17] J. Babič , B. Likozar , A. Pavko , Int. J. Mol. Sci. 2012, 13, 11365. 23109859 10.3390/ijms130911365 PMC3472751

[18] D. Areskogh , J. Li , P. Nousiainen , G. Gellerstedt , J. Sipilä , G. Henriksson , Holzforschung 2010, 64, 21.

[19] P. Widsten , A. Kandelbauer , Enzyme Microb. Technol. 2008, 42, 293.

[20] M. M. Cajnko , J. Oblak , M. Grilc , B. Likozar , Bioresour. Technol. 2021, 340, 125655. 34388661 10.1016/j.biortech.2021.125655

[21] Z. Li , J. Zhang , L. Qin , Y. Ge , ACS Sustain. Chem. Eng. 2018, 6, 2591.

[22] M. L. Mattinen , J. J. Valle‐Delgado , T. Leskinen , T. Anttila , G. Riviere , M. Sipponen , A. Paananen , K. Lintinen , M. Kostiainen , M. Österberg , Enzyme Microb. Technol. 2018, 111, 48. 29421036 10.1016/j.enzmictec.2018.01.005

[23] P. Figueiredo , D. Morais de Carvalho , M. H. Lahtinen , K. S. Hilden , K. S. Mikkonen , Sustainable Mater. Technol. 2023, 37, e00677.

[24] H. Nsairat , Z. Lafi , M. Al‐Sulaibi , L. Gharaibeh , W. Alshaer , Food Chem. 2023, 424, 136438. 37244187 10.1016/j.foodchem.2023.136438

[25] M. Plaza‐Oliver , M. J. Santander‐Ortega , M. V. Lozano , Drug Delivery Transl. Res. 2021, 11, 471. 10.1007/s13346-021-00908-7 PMC7852471 33528830

[26] A. D. Kulkarni , A. A. Joshi , C. L. Patil , P. D. Amale , H. M. Patel , S. J. Surana , V. S. Belgamwar , K. S. Chaudhari , C. V. Pardeshi , Int. J. Biol. Macromol. 2017, 104, 799. 28648637 10.1016/j.ijbiomac.2017.06.088

[27] E. S. Wibowo , B. D. Park , Wood Sci. Technol. 2022, 56, 1047.

[28] S. Imlimthan , P. Figueiredo , H. A. Santos , M. Sarparanta , Lignin‐Based Materials for Biomedical Applications, Elsevier 2021, 1.

[29] F. Abik , C. Palasingh , M. Bhattarai , S. Leivers , A. Ström , B. Westereng , K. S. Mikkonen , T. Nypelö , J. Agric. Food Chem. 2023, 71, 2667. 36724217 10.1021/acs.jafc.2c06449 PMC9936590

[30] H. Sixta , Handbook of Pulp 2008, 1–2, Wiley, 1.

[31] T. M. Ho , F. Abik , S. Hietala , E. Isaza Ferro , L. Pitkänen , D. W. Juhl , T. Vosegaard , P. O. Kilpeläinen , K. S. Mikkonen , Cellulose 2023, 30, 753.

[32] D. M. De Carvalho , M. H. Lahtinen , M. Lawoko , K. S. Mikkonen , ACS Sustain. Chem. Eng. 2020, 8, 11795.

[33] P. Figueiredo , M. H. Lahtinen , M. B. Agustin , D. M. de Carvalho , S. P. Hirvonen , P. A. Penttilä , K. S. Mikkonen , ChemSusChem. 2021, 14, 4718. 34398512 10.1002/cssc.202101356 PMC8596756

[34] W. Zhao , L. P. Xiao , G. Song , R. C. Sun , L. He , S. Singh , B. A. Simmons , G. Cheng , Green Chem. 2017, 19, 3272.

[35] L. Wang , K. Shigetomi , K. Koda , A. Gele , Y. Uraki , Holzforschung 2020, 74, 551.

[36] I. V. Pylypchuk , M. Karlsson , P. A. Lindén , M. E. Lindström , T. Elder , O. Sevastyanova , M. Lawoko , Green Chem. 2023, 25, 4415. 37288453 10.1039/d3gc00703k PMC10243429

[37] M. J. Suota , T. A. da Silva , S. F. Zawadzki , G. L. Sassaki , F. A. Hansel , M. Paleologou , L. P. Ramos , Ind. Crops Prod. 2021, 173, 114138.

[38] A. Moreno , J. Liu , M. Morsali , M. H. Sipponen , Micro and Nanolignin in Aqueous Dispersions and Polymers, Elsevier 2022, 385.

[39] D. Geißler , N. Nirmalananthan‐Budau , L. Scholtz , I. Tavernaro , U. Resch‐Genger , Microchim. Acta 2021, 188, 321. 10.1007/s00604-021-04960-5 PMC8418596 34482449

[40] L. Guerrini , R. A. Alvarez‐Puebla , N. Pazos‐Perez , Materials 2018, 11, 1154. 29986436 10.3390/ma11071154 PMC6073273

[41] M. H. Sipponen , H. Lange , M. Ago , C. Crestini , ACS Sustain. Chem. Eng. 2018, 6, 9342. 30271691 10.1021/acssuschemeng.8b01652 PMC6156105

[42] M. Österberg , K. A. Henn , M. Farooq , J. J. Valle‐Delgado , Chem. Rev. 2023, 123, 2200. 36720130 10.1021/acs.chemrev.2c00492 PMC9999428

[43] T. Benselfelt , E. D. Cranston , S. Ondaral , E. Johansson , H. Brumer , M. W. Rutland , L. Wågberg , Biomacromolecules 2016, 17, 2801. 27476615 10.1021/acs.biomac.6b00561

[44] M. S. Reid , M. Villalobos , E. D. Cranston , Curr. Opin. Colloid Interface Sci. 2017, 29, 76.

[45] M. Q. Ai , F. F. Wang , F. Huang , J. Microbiol. Biotechnol. 2015, 25, 1361. 25876603 10.4014/jmb.1502.02022

[46] L. Munk , M. L. Andersen , A. S. Meyer , Enzyme Microb. Technol. 2018, 116, 48. 29887016 10.1016/j.enzmictec.2018.05.009

[47] V. Perna , A. S. Meyer , J. Holck , L. D. Eltis , V. G. H. Eijsink , J. Wittrup Agger , ACS Sustain. Chem. Eng. 2020, 8, 831.

[48] B. Ahvazi , É. Cloutier , O. Wojciechowicz , T. D. Ngo , ACS Sustain. Chem. Eng. 2016, 4, 5090.

[49] J. L. Wen , L. P. Xiao , Y. C. Sun , S. N. Sun , F. Xu , R. C. Sun , X. L. Zhang , Carbohydr. Res. 2011, 346, 111. 21109235 10.1016/j.carres.2010.10.006

[50] D. K. Shen , S. Gu , A. V. Bridgwater , Carbohydr. Polym. 2010, 82, 39.

[51] M. González Martínez , T. Ohra‐aho , D. da Silva Perez , T. Tamminen , C. Dupont , J. Anal. Appl. Pyrolysis 2019, 137, 195.

[52] M. Balakshin , E. Capanema , A. Berlin , Stud. Nat. Prod. Chem. 2014, 42, 83.

[53] D. Tarasov , M. Leitch , P. Fatehi , Biotechnol. Biofuels 2018, 11, 269. 30288174 10.1186/s13068-018-1262-1 PMC6162904

[54] P. Oinonen , L. Zhang , M. Lawoko , G. Henriksson , Phytochemistry 2015, 111, 177. 25549980 10.1016/j.phytochem.2014.10.027

[55] N. Takahashi , T. Koshijima , Wood Sci. Technol. 1988, 22, 231.

[56] T. Kondo , K. Mizuno , T. Kato , T. Hiroi , Can. J. Plant Sci. 1990, 70, 193.

[57] T. Saarinen , H. Orelma , S. Grönqvist , M. Andberg , S. Holappa , J. Laine , Bioresources 2009, 4, 94.

[58] M. Fernández‐Fernández , M. A. Sanromán , D. Moldes , Wood Sci. Technol. 2014, 48, 151.

[59] R. Rasch , A. Stricher , R. W. Truss , J. Appl. Polym. Sci. 2014, 131, 39572.

[60] T. Ghosh , T. Elo , V. S. Parihar , P. Maiti , R. Layek , Ind. Crops. Prod. 2022, 187, 115299.

[61] N. Ghavidel , M. K. R. Konduri , P. Fatehi , Ind. Crops. Prod. 2021, 172, 113950.

[62] D. M. D. Carvalho , M. H. Lahtinen , M. Bhattarai , M. Lawoko , K. S. Mikkonen , Green Chem. 2021, 23, 9084.

[63] M. Farooq , T. Zou , G. Riviere , M. H. Sipponen , M. Österberg , Biomacromolecules 2019, 20, 693. 30358992 10.1021/acs.biomac.8b01364

[64] D. Areskogh , J. Li , G. Gellerstedt , G. Henriksson , Biomacromolecules 2010, 11, 904. 20175586 10.1021/bm901258v

[65] A. Sundberg , K. Sundberg , C. Lillandt , B. Holmbom , Nord. Pulp. Paper. Res. J. 1996, 11, 216.

