# Supplementary material for: Spontaneous and Laccase‐Assisted Adsorption of Hemicelluloses Rendering Colloidal Stability of Lignin Nanoparticles at Low pH
Source: ChemSusChem. 2025 May 30;18(14):e202500668. doi: 10.1002/cssc.202500668 (PMC12270363; doi:10.1002/cssc.202500668)
Supplement: Supplementary file 1 — Supplementary Material [file CSSC-18-e202500668-s001.pdf]

Supporting information

Spontaneous and laccase-assisted adsorption of hemicelluloses render colloidal stability of lignin nanoparticles at low pH

Patrícia Figueiredo[a,*], Danila Morais de Carvalho[a], Kristiina S. Hilden[b], Maarit Lahtinen[a], and Kirsi S. Mikkonen[a,c,*]

[a] Department of Food and Nutrition, P.O. Box 66, FI-00014, University of Helsinki, Finland

[b] Department of Microbiology, Faculty of Agriculture and Forestry, FI-00014, University of Helsinki, Finland

[c] Helsinki Institute of Sustainability Science (HELSUS), FI-00014, University of Helsinki, Finland

Corresponding author: patricia.figueiredo@helsinki.fi; kirsi.s.mikkonen@helsinki.fi

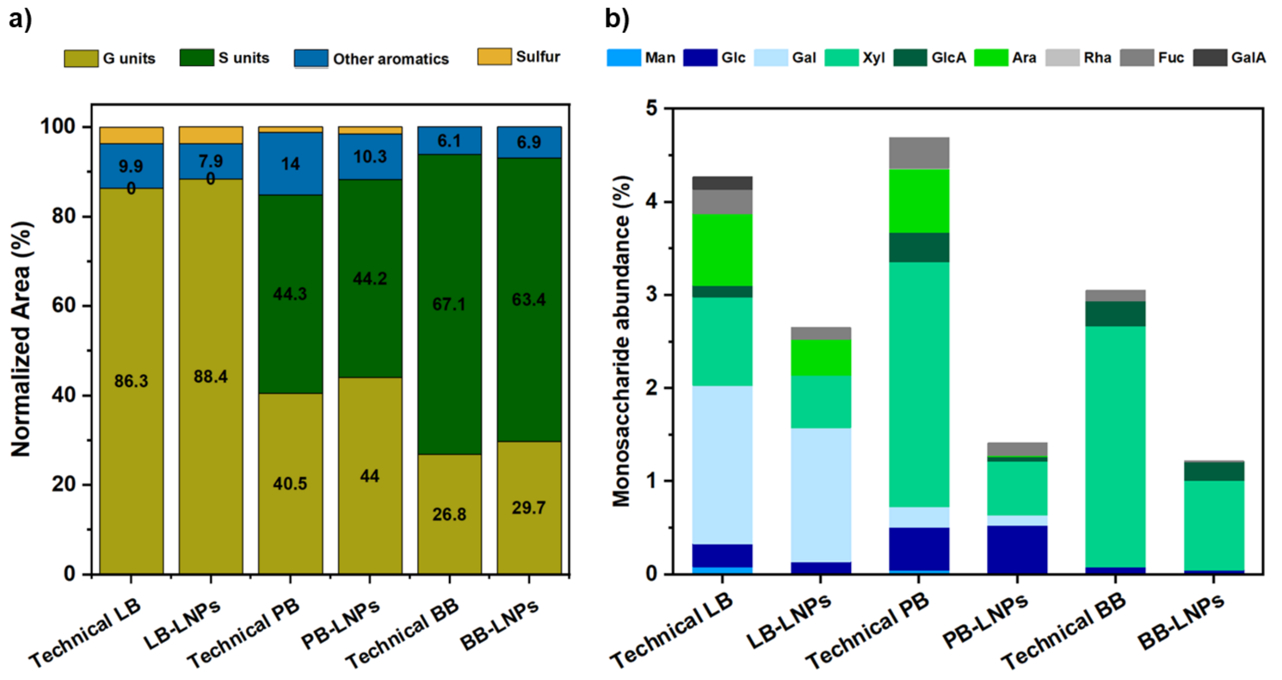

Figure S1. Representation of **a)** pyrolysis GCMS and **b)** monosaccharides composition of the technical lignins and respective LNPs after preparation.

Table S1. Monosaccharides composition of the technical lignins and respective LNPs after preparation.

Sample	Monosaccharides abundance (%)									
	Man	Glc	Gal	Xyl	GlcA	Ara	Rha	Fuc	GalA	Total
**Technical LB**	0.08 a	0.25 a	1.70 a	0.95 a	0.13 a	0.77 a	n.d.	0.26 a	0.13 a	**4.26 a**
**LB-LNPs (stock)**	0.00 a	0.14 b	1.44 b	0.56 b	0.00 b	0.39 b	n.d.	0.12 a	0.00 a	**2.65 b**
**Technical PB**	0.05 a	0.46 a	0.22 a	2.63 a	0.32 a	0.68 a	0.01 a	0.32 a	n.d.	**4.69 a**
**PB-LNPs (stock)**	0.00 b	0.53 a	0.11 b	0.58 b	0.05 b	0.01 b	0.00 a	0.13 a	n.d.	**1.41 b**
**Technical BB**	n.d.	0.08 a	n.d.	2.59 a	0.27 a	n.d.	n.d.	0.11 a	n.d.	**3.06 a**
**BB-LNPs (stock)**	n.d.	0.05 a	n.d.	0.96 b	0.20 b	n.d.	n.d.	0.01 a	n.d.	**1.22 b**

Notes: For the same lignin type the averages in the column followed by the same letter do not differ from each other at a probability level of 5% according to Tukey's test. **Abbreviations:** arabinose (Ara), fucose (Fuc), galactose (Gal), galacturonic acid (GalA), glucose (Glc), glucuronic acid (GlcA), mannose (Man), not detected (n.d.), rhamnose (Rha), and xylose (Xyl).

Table S2. Characterization of the different water-soluble fraction of hemicelluloses used for the reactions.

	**sdGGM**	**epGGM**	**epBLN-GGM**	**sdGX**	**epGX**
**DLS (in MQ-water)**					
**ζ-potential (mV)**	-21.8	-18.0	-19.9	-21.6	-16.4
**Py-GCMS**					
**Hemicellulose (%)**	85.5	95.9	98.7	81.7	87.1
**Lignin (%)**	**14.4**	**4.1**	**1.3**	**18.3**	**12 .9**
**G units (%)**	13.0	4.1	1.3	2.2	0.8
**S units (%)**	-	-	-	16.1	10.8
**Other aromatics (%)**	1.3	-	-	-	1.3
**Acid Methanolysis**					
**Man (%)**	51.58	60.73	64.14	0.90	2.38
**Glc (%)**	12.31	13.92	17.07	0.00	0.00
**Gal (%)**	5.87	7.50	12.18	0.00	0.00
**Xyl (%)**	8.47	11.02	5.78	91.55	95.01
**GlcA (%)**	n.d.	n.d.	n.d.	n.d.	n.d.
**Ara (%)**	n.d.	n.d.	n.d.	n.d.	n.d.
**Rha (%)**	n.d.	n.d.	n.d.	n.d.	n.d.
**Fuc (%)**	n.d.	n.d.	n.d.	n.d.	n.d.
**GalA (%)**	n.d.	n.d.	n.d.	n.d.	n.d.
**Total (%)**	78.23	93.16	99.17	92.45	97.39

Abbreviations: arabinose (Ara), fucose (Fuc), galactose (Gal), galacturonic acid (GalA), glucose (Glc), glucuronic acid (GlcA), mannose (Man), not detected (n.d.), rhamnose (Rha), and xylose (Xyl).

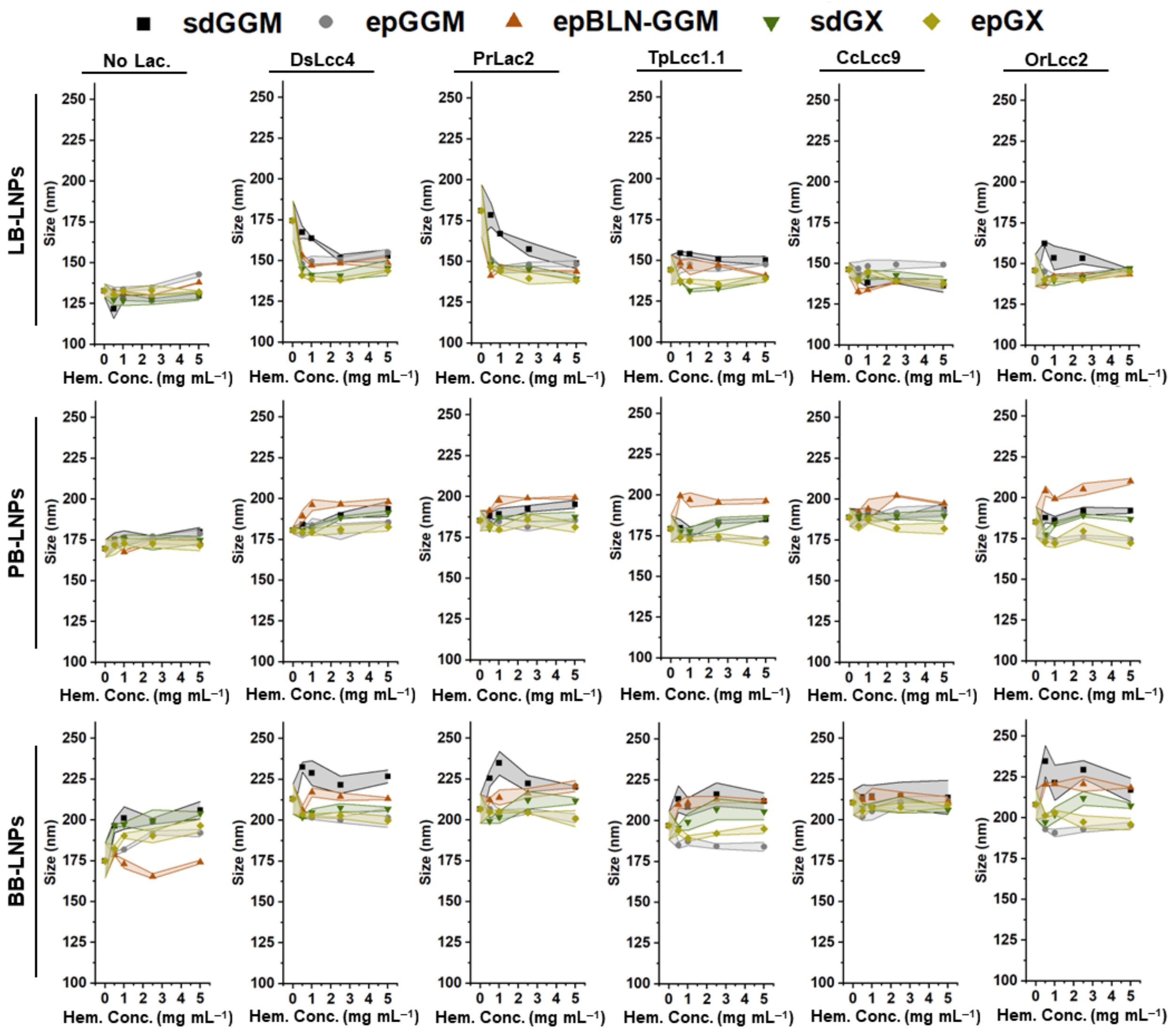

Figure S2. Size of the LNPs measured by dynamic light scattering after incubation of LNPs with the different laccases (1000 nKat g^{-1} of lignin) and hemicelluloses (0.5–5 mg mL^{-1}), at pH 5 and RT for 24 h, and stored for 7 weeks at 4 °C. The lines between the symbols are to guide the eye, and their width represents the s.d. of each measured point (n ≥ 3).

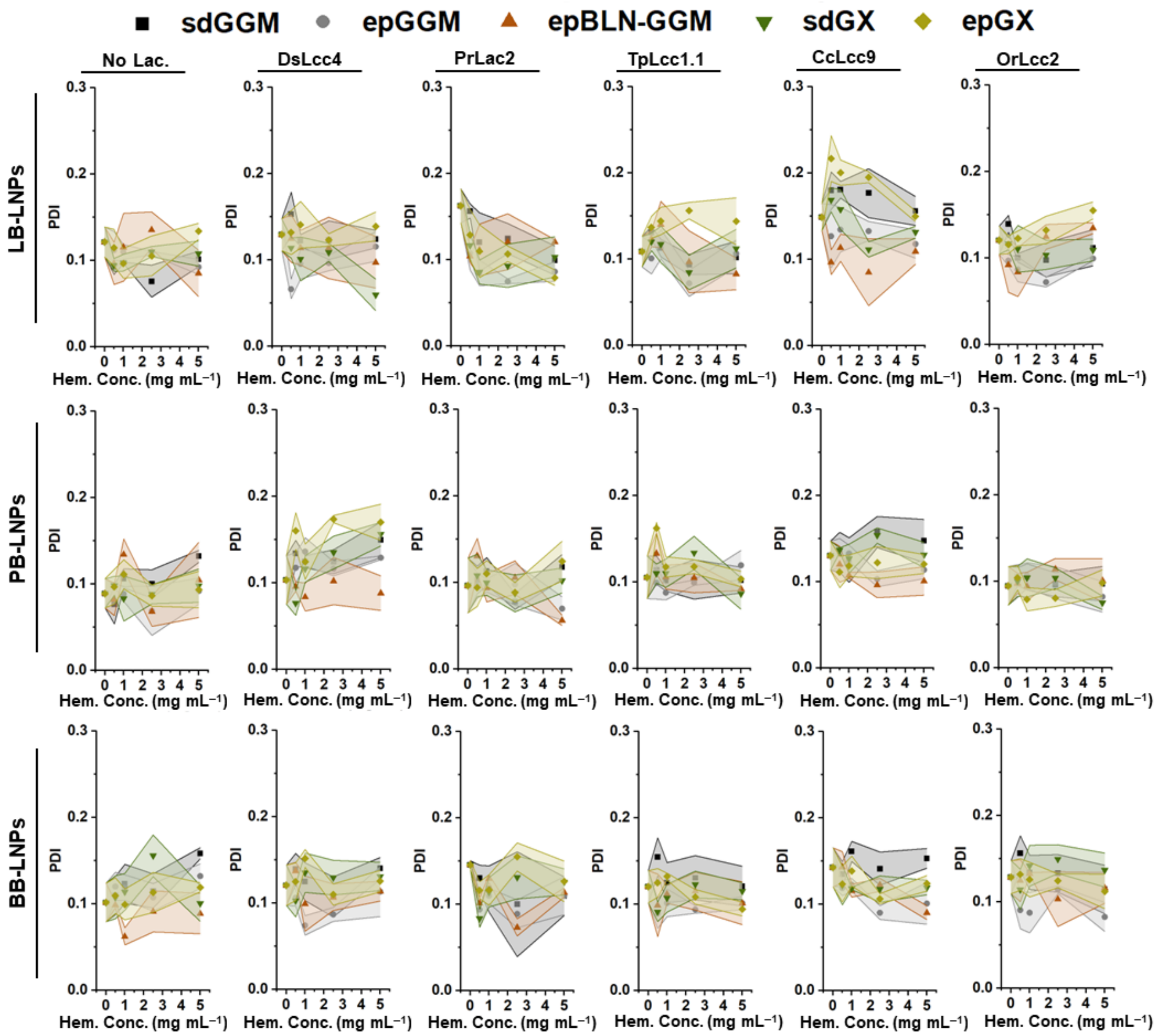

Figure S3. Polydispersity index (PDI) of LNPs measured by dynamic light scattering after incubation of LNPs with the different laccases (1000 nKat g^{-1} of lignin) and hemicelluloses (0.5–5 mg mL^{-1}), at pH 5 and RT for 24 h. The lines between the symbols are to guide the eye, and their width represents the s.d. of each measured point (n ≥ 3).

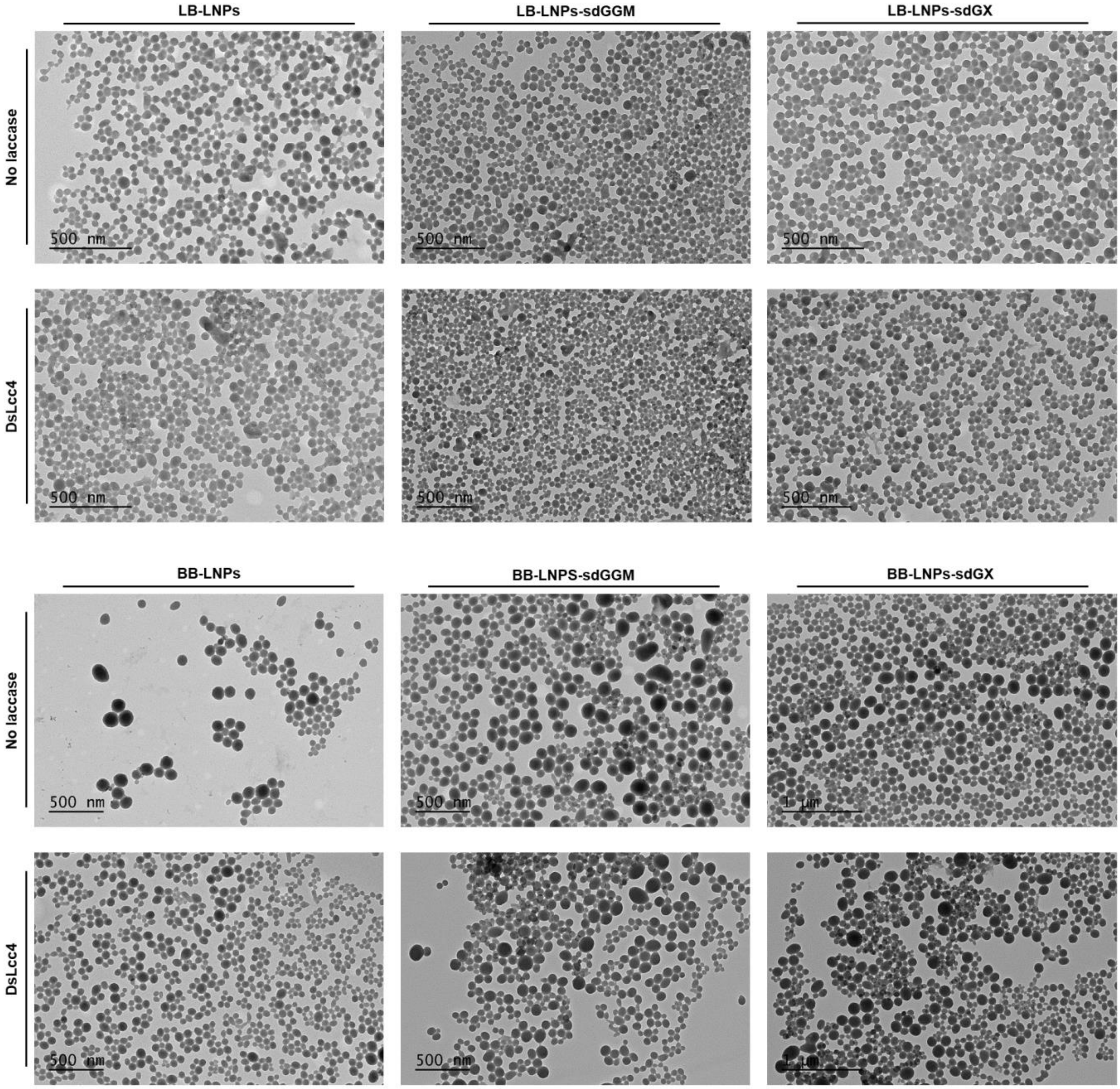

Figure S4. Representative TEM pictures of LB- and BB-LNPs treated with DsLcc4 and sdGGM or sdGX, and respective controls.

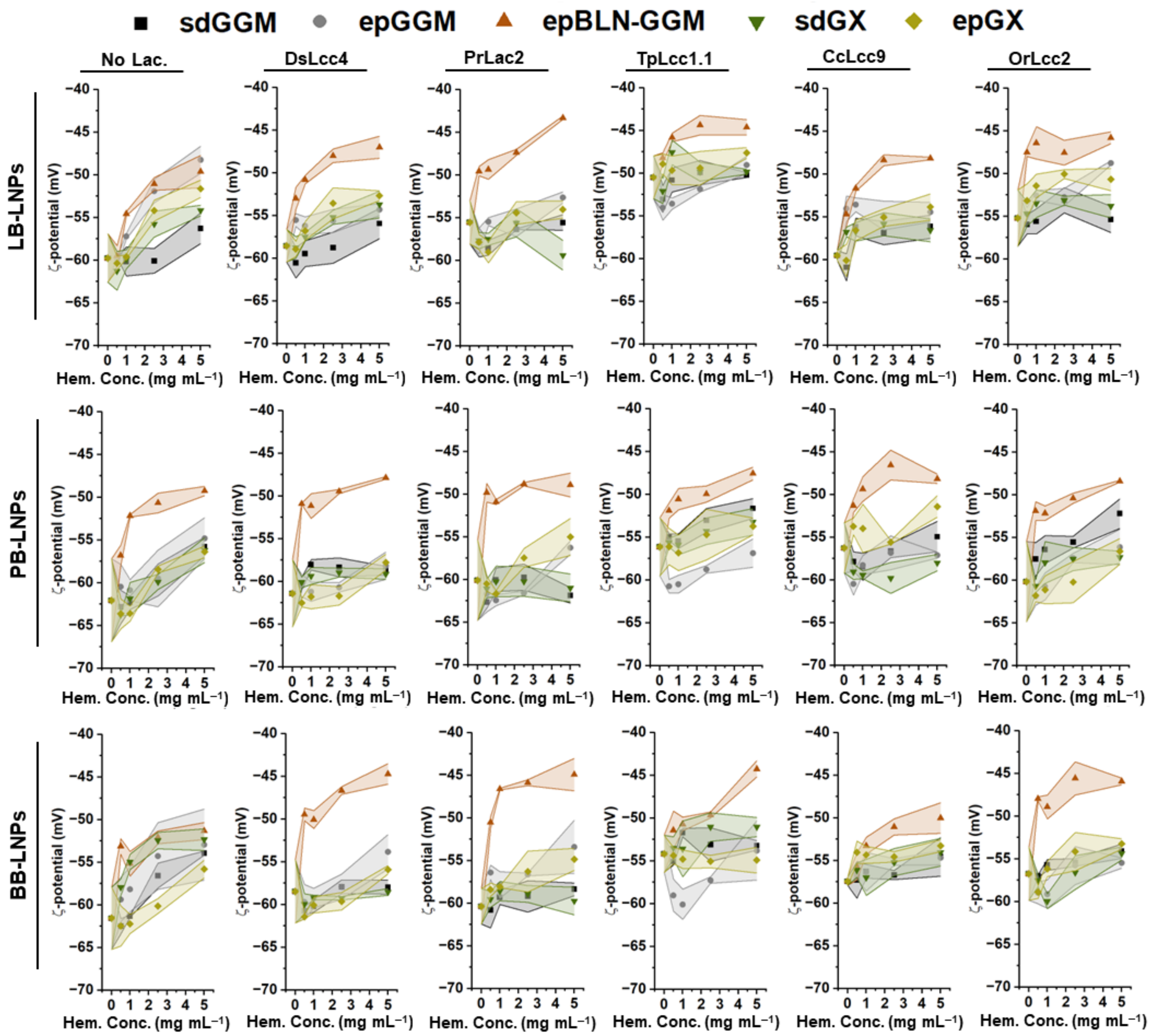

Figure S5. Surface charge of LNPs, given by the ζ-potential, measured by dynamic light scattering after incubation of LNPs with the different laccases (1000 nKat g^{-1} of lignin) and hemicelluloses (0.5–5 mg mL^{-1}), at pH 5 and RT for 24 h, and stored for 7 weeks at 4 °C. The lines between the symbols are to guide the eye, and their width represents the s.d. of each measured point (n ≥ 3).

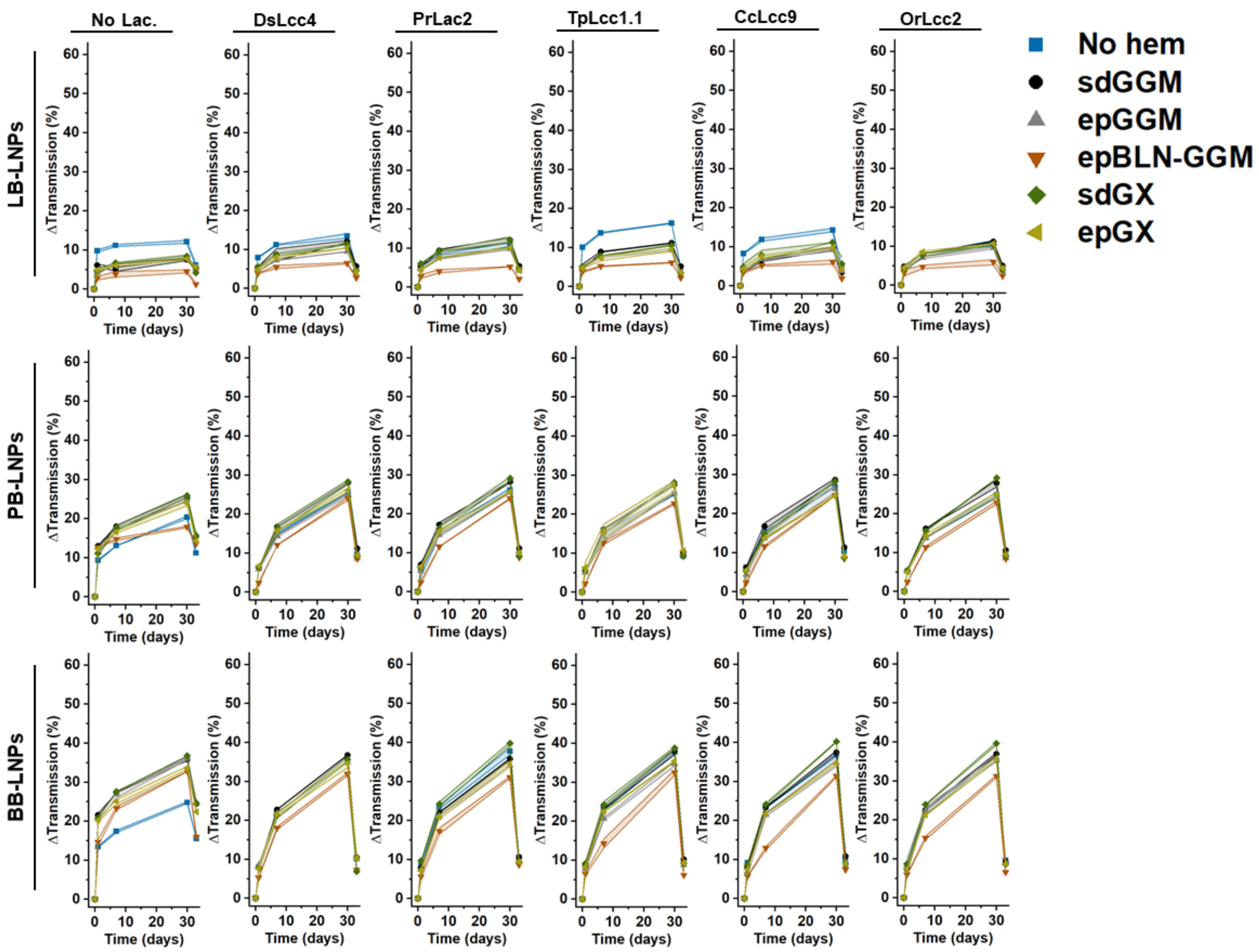

Figure S6. Colloidal stability of LNPs in terms of variation in the transmitted light through the LNP suspensions in MilliQ-water, evaluated using Turbiscan. The LNPs were previously incubated with the different laccases (1000 nKat g^{-1} of lignin) and hemicelluloses (2.5 mg mL^{-1}), at pH 5 and RT for 24 h. The lines between the symbols are to guide the eye, and their width represents the s.d. of each measured point (n = 2).

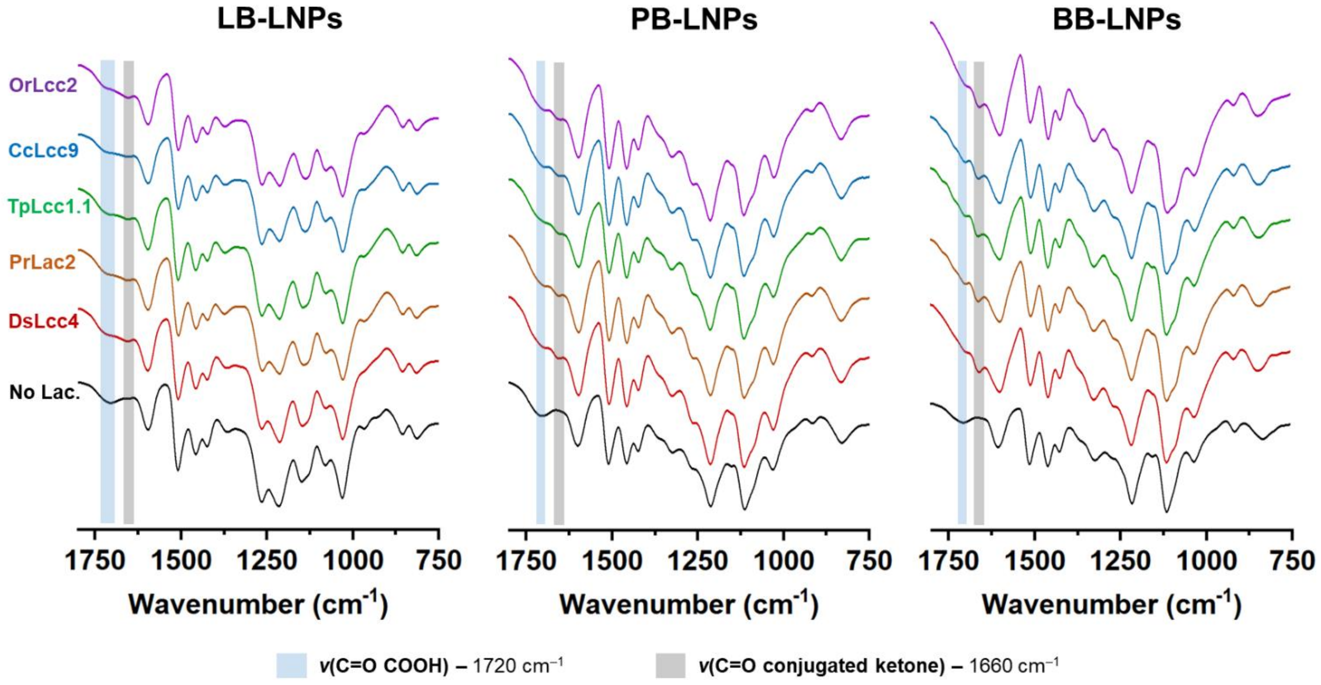

Figure S7. ATR-FTIR spectra of LNPs before (no lac.) and after incubation with the different laccases (1000 nKat g^{-1} of lignin), at pH 5 and RT for 24 h.

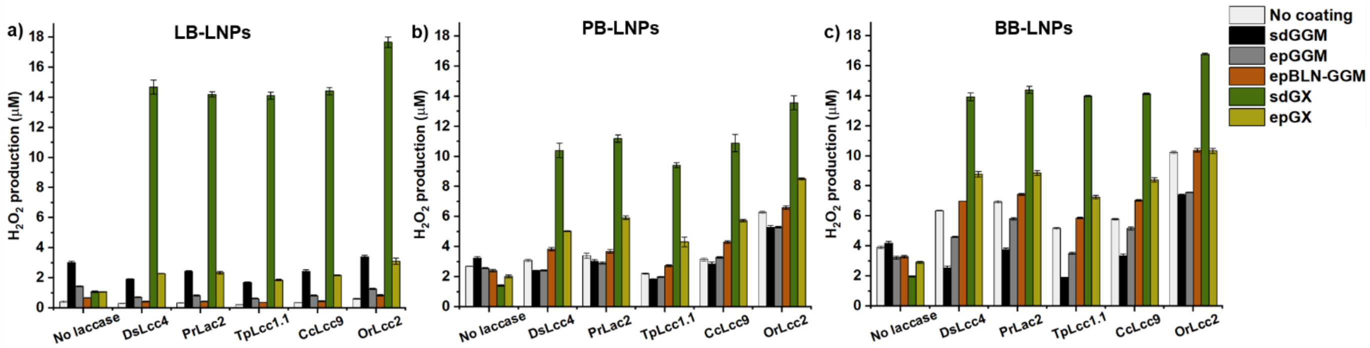

Figure S8. Quantification of the *in situ* H_2O_2 production after incubation of **a)** LB-LNPs (softwood), **b)** PB-LNPs (grass), and **c)** BB-LNPs (hardwood) with the different laccases (1000 nKat g^{-1} of lignin) and hemicelluloses (2.5 mg mL^{-1}), at pH 5 and RT for 1 h, using the Amplex™ Red Hydrogen Peroxide/Peroxidase Assay Kit. Error bars represent the s.d. of each measured point (n = 3).

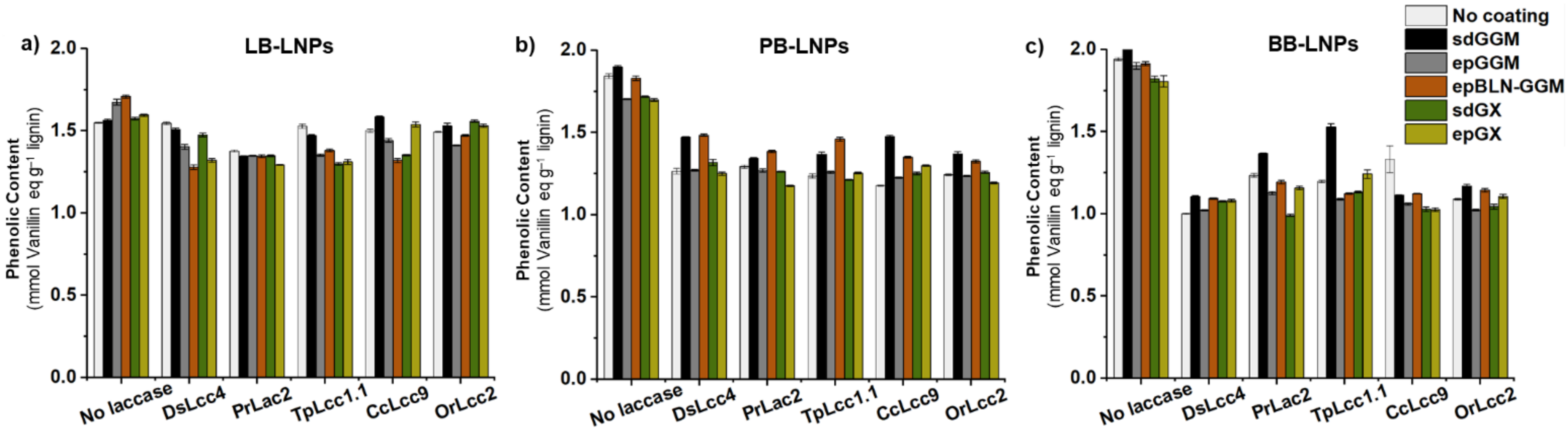

Figure S9. Quantification of the phenolic content on **a)** LB-LNPs, **b)** PB-LNPs, and **c)** BB-LNPs, after their incubation with the different laccases (1000 nKat g^{-1} of lignin) and hemicelluloses (2.5 mg mL^{-1}), at pH 5 and RT for 24 h, using the Amplex™ Red Hydrogen Peroxide/Peroxidase Assay Kit. Control samples were also analyzed in the absence of laccase and/or hemicelluloses. Error bars represent the s.d. of each measured point (n = 3).

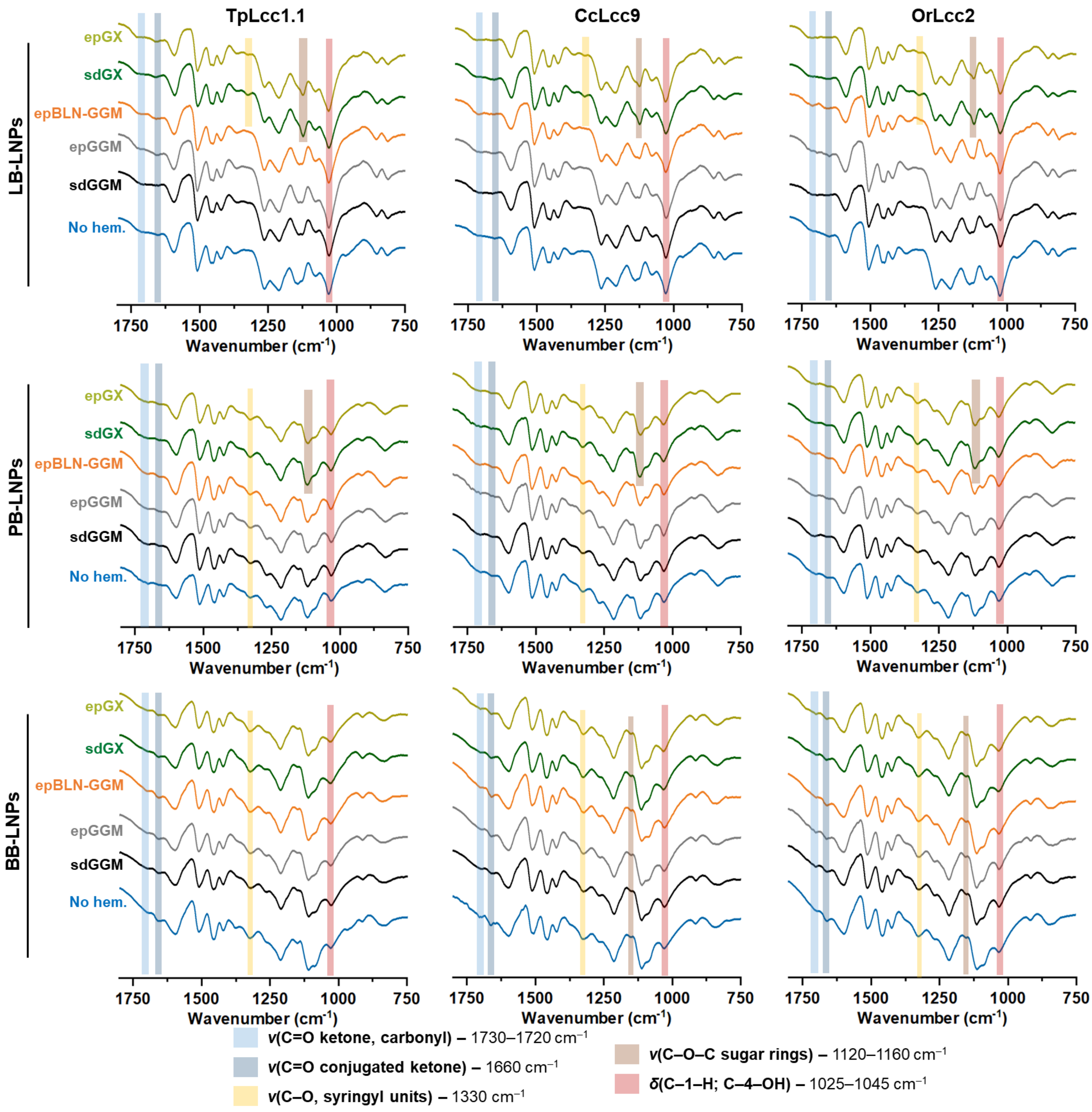

Figure S10. ATR-FTIR spectra of LNPs, after their incubation with the different laccases (1000 nKat g^{-1} of lignin) and hemicelluloses (2.5 mg mL^{-1}), at pH 5 and RT for 24 h.

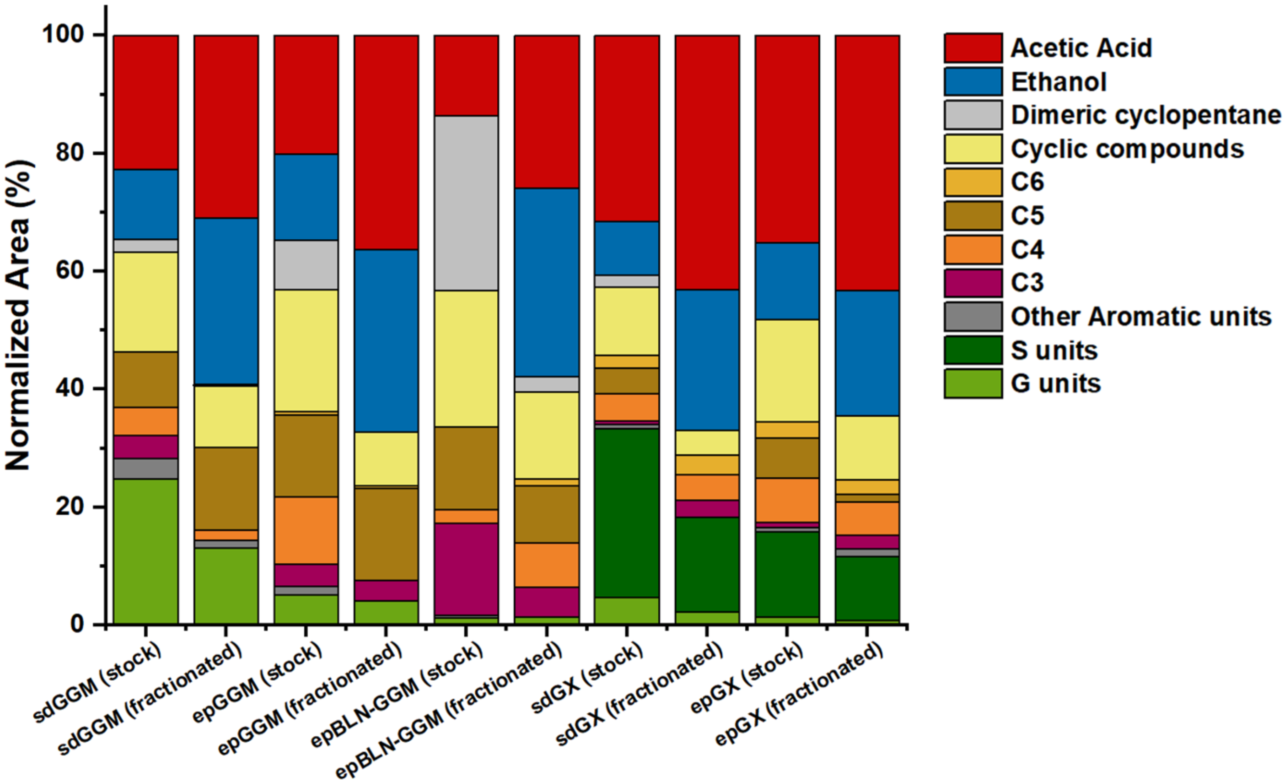

Figure S11. Py-GCMS analysis of the different hemicelluloses, before (stock) and after fractionation (water-soluble fraction).

Table S3. Area (%) of the lignin-derived compounds identified by Py-GCMS analysis, after reaction of LB-LNPs with hemicelluloses and laccases.

Treatment	Hemicel. (%)	G units (%)	S units (%)	Other Aromatic units (%)	Sulfur (%)
**No laccase – No hem**	0.00	88.97	0.00	7.34	3.69
**No laccase – sdGGM**	0.00	88.33	0.00	8.90	2.78
**No laccase – epGGM**	0.00	87.86	0.00	8.53	3.61
**No laccase – epBLN-GGM**	0.42	89.91	0.00	5.96	3.72
**No laccase – sdGX**	1.40	78.82	7.20	9.48	3.10
**No laccase – epGX**	1.31	77.42	9.49	8.56	3.22
***Ds*Lcc4 – No hem**	0.00	91.27	0.00	4.37	4.36
***Ds*Lcc4 – sdGGM**	0.33	89.66	0.00	6.48	3.54
***Ds*Lcc4 – epGGM**	1.23	87.30	0.00	7.57	3.89
***Ds*Lcc4 – epBLN-GGM**	0.57	89.63	0.00	5.67	3.84
***Ds*Lcc4 – sdGX**	1.44	70.80	18.37	5.50	3.89
***Ds*Lcc4 – epGX**	1.73	78.57	7.03	8.41	4.26
***Pr*Lac2 – No hem**	0.00	88.43	0.00	7.08	4.50
***Pr*Lac2 – sdGGM**	0.00	86.96	0.00	8.47	4.57
***Pr*Lac2 – epGGM**	1.11	88.39	0.00	6.69	3.81
***Pr*Lac2 – epBLN-GGM**	1.13	86.67	0.00	8.16	4.05
***Pr*Lac2 – sdGX**	1.20	70.34	19.78	5.05	3.62
***Pr*Lac2 – epGX**	1.80	78.78	9.28	5.34	4.80
***Tp*Lcc1.1 – No hem**	0.00	89.65	0.00	6.38	3.98
***Tp*Lcc1.1 – sdGGM**	0.00	86.31	0.00	9.66	4.03
***Tp*Lcc1.1 – epGGM**	0.00	89.23	0.00	6.48	4.30
***Tp*Lcc1.1 – epBLN-GGM**	0.83	88.43	0.00	6.26	4.48
***Tp*Lcc1.1 – sdGX**	1.39	69.44	19.32	5.56	4.28
***Tp*Lcc1.1 – epGX**	2.24	77.43	8.51	7.17	4.65
***Cc*Lcc9 – No hem**	0.00	86.51	0.00	8.92	4.57
***Cc*Lcc9 – sdGGM**	0.45	87.50	0.00	8.20	3.86
***Cc*Lcc9 – epGGM**	0.76	85.15	0.00	9.68	4.42
***Cc*Lcc9 – epBLN-GGM**	0.72	88.42	0.00	6.32	4.55
***Cc*Lcc9 – sdGX**	1.68	70.87	15.88	7.43	4.15
***Cc*Lcc9 – epGX**	2.10	76.26	10.75	6.59	4.30
***Or*Lcc2 – No hem**	0.00	87.79	0.00	7.44	4.77
***Or*Lcc2 – sdGGM**	0.00	85.59	0.00	9.78	4.63
***Or*Lcc2 – epGGM**	0.00	89.09	0.00	6.50	4.41
***Or*Lcc2 – epBLN-GGM**	0.44	89.19	0.00	5.65	4.71
***Or*Lcc2 – sdGX**	2.50	69.77	13.76	10.17	3.80
***Or*Lcc2 – epGX**	1.98	79.01	9.64	5.69	3.68

Table S4. Area (%) of the lignin-derived compounds identified by Py-GCMS analysis, after reaction of PB-LNPs with hemicelluloses and laccases.

Treatment	Hemicel. (%)	G units (%)	S units (%)	Other Aromatic units (%)	Sulfur (%)
**No laccase – No hem**	0.00	38.66	36.30	23.81	1.24
**No laccase – sdGGM**	0.62	42.89	37.01	18.28	1.20
**No laccase – epGGM**	0.31	41.07	36.66	20.94	1.03
**No laccase – epBLN-GGM**	0.84	42.26	37.01	18.68	1.22
**No laccase – sdGX**	0.84	37.91	37.22	22.89	1.14
**No laccase – epGX**	1.23	37.69	36.29	23.71	1.09
***Ds*Lcc4 – No hem**	0.00	38.52	34.91	25.94	1.33
***Ds*Lcc4 – sdGGM**	0.96	42.99	32.39	22.39	1.27
***Ds*Lcc4 – epGGM**	0.95	41.16	34.81	21.61	1.46
***Ds*Lcc4 – epBLN-GGM**	0.72	42.03	37.60	18.05	1.60
***Ds*Lcc4 – sdGX**	1.50	35.52	41.08	20.56	1.33
***Ds*Lcc4 – epGX**	1.93	37.97	37.68	20.75	1.68
***Pr*Lac2 – No hem**	0.00	40.37	37.46	20.7	1.47
***Pr*Lac2 – sdGGM**	0.83	42.10	35.24	20.33	1.50
***Pr*Lac2 – epGGM**	0.00	41.97	35.32	21.02	1.69
***Pr*Lac2 – epBLN-GGM**	0.64	41.50	36.90	19.55	1.41
***Pr*Lac2 – sdGX**	1.20	36.10	40.47	20.92	1.31
***Pr*Lac2 – epGX**	1.97	38.45	37.79	20.51	1.27
***Tp*Lcc1.1 – No hem**	0.00	39.27	36.23	22.97	1.53
***Tp*Lcc1.1 – sdGGM**	0.67	44.93	32.57	20.63	1.22
***Tp*Lcc1.1 – epGGM**	0.83	41.59	34.32	21.87	1.40
***Tp*Lcc1.1 – epBLN-GGM**	0.79	41.42	36.23	19.56	2.00
***Tp*Lcc1.1 – sdGX**	1.52	33.98	41.93	21.19	1.38
***Tp*Lcc1.1 – epGX**	1.61	37.61	39.87	19.5	1.41
***Cc*Lcc9 – No hem**	0.00	39.88	39.16	19.83	1.13
***Cc*Lcc9 – sdGGM**	0.61	42.54	34.82	20.69	1.34
***Cc*Lcc9 – epGGM**	0.67	41.41	36.77	19.79	1.37
***Cc*Lcc9 – epBLN-GGM**	0.59	41.56	37.51	19.09	1.26
***Cc*Lcc9 – sdGX**	1.84	35.26	44.57	17.05	1.27
***Cc*Lcc9 – epGX**	1.78	37.42	42.16	17.13	1.50
***Or*Lcc2 – No hem**	0.00	42.19	38.43	17.79	1.59
***Or*Lcc2 – sdGGM**	0.28	46.51	36.72	14.98	1.51
***Or*Lcc2 – epGGM**	0.64	41.97	40.30	15.4	1.69
***Or*Lcc2 – epBLN-GGM**	1.85	42.94	34.88	18.89	1.43
***Or*Lcc2 – sdGX**	2.42	36.27	44.95	14.72	1.62
***Or*Lcc2 – epGX**	1.49	38.82	41.70	16.74	1.25

Table S5. Area (%) of the lignin-derived compounds identified by Py-GCMS analysis, after reaction of BB-LNPs with hemicelluloses and laccases.

Treatment	Hemicel. (%)	G units (%)	S units (%)	Other Aromatic units (%)	Sulfur (%)
**No laccase – No hem**	0.00	29.72	63.35	6.93	0.00
**No laccase – sdGGM**	0.54	32.46	61.73	5.27	0.00
**No laccase – epGGM**	0.51	33.85	60.29	5.35	0.00
**No laccase – epBLN-GGM**	1.00	30.80	65.22	2.98	0.00
**No laccase – sdGX**	1.10	29.83	64.56	4.50	0.00
**No laccase – epGX**	1.13	29.43	65.74	3.70	0.00
***Ds*Lcc4 – No hem**	0.00	34.04	62.14	3.83	0.00
***Ds*Lcc4 – sdGGM**	2.04	38.04	58.76	1.15	0.00
***Ds*Lcc4 – epGGM**	1.49	34.74	57.97	5.79	0.00
***Ds*Lcc4 – epBLN-GGM**	2.17	33.81	61.90	2.12	0.00
***Ds*Lcc4 – sdGX**	0.46	26.78	70.66	2.11	0.00
***Ds*Lcc4 – epGX**	2.45	28.87	65.29	3.39	0.00
***Pr*Lac2 – No hem**	0.00	34.17	60.91	4.92	0.00
***Pr*Lac2 – sdGGM**	0.55	37.08	57.21	5.16	0.00
***Pr*Lac2 – epGGM**	0.78	33.49	60.71	5.02	0.00
***Pr*Lac2 – epBLN-GGM**	1.01	32.93	62.90	3.16	0.00
***Pr*Lac2 – sdGX**	1.63	28.13	67.55	2.69	0.00
***Pr*Lac2 – epGX**	2.41	27.06	68.17	2.36	0.00
***Tp*Lcc1.1 – No hem**	0.00	33.48	60.97	5.09	0.00
***Tp*Lcc1.1 – sdGGM**	1.01	38.03	56.59	4.37	0.00
***Tp*Lcc1.1 – epGGM**	2.10	30.51	65.19	2.20	0.00
***Tp*Lcc1.1 – epBLN-GGM**	1.17	31.95	63.60	3.28	0.00
***Tp*Lcc1.1 – sdGX**	1.37	27.90	68.15	2.58	0.00
***Tp*Lcc1.1 – epGX**	3.00	28.69	64.70	3.61	0.00
***Cc*Lcc9 – No hem**	0.00	30.60	67.13	2.27	0.00
***Cc*Lcc9 – sdGGM**	0.00	38.49	54.49	7.02	0.00
***Cc*Lcc9 – epGGM**	0.98	35.90	58.88	4.25	0.00
***Cc*Lcc9 – epBLN-GGM**	1.01	31.74	63.63	3.62	0.00
***Cc*Lcc9 – sdGX**	1.13	27.88	69.08	1.91	0.00
***Cc*Lcc9 – epGX**	2.45	29.59	65.35	2.62	0.00
***Or*Lcc2 – No hem**	0.00	33.85	60.54	4.84	0.00
***Or*Lcc2 – sdGGM**	0.67	37.99	54.52	6.81	0.00
***Or*Lcc2 – epGGM**	1.18	35.40	60.41	3.01	0.00
***Or*Lcc2 – epBLN-GGM**	1.51	31.65	64.71	2.14	0.00
***Or*Lcc2 – sdGX**	1.69	27.63	66.30	4.38	0.00
***Or*Lcc2 – epGX**	2.20	29.25	65.83	2.71	0.00

Table S6. Monosaccharide composition of LNPs coated with the different hemicelluloses (*i.e.*, sdGGM, epGGM, BLN GGM, sdGX, and epGX) by spontaneous adsorption (no laccase) or *Ds*Lcc4 treatment.

LNPs	Hemicellulose coating	Monosaccharides abundance (%)									
		Man	Glc	Gal	Xyl	GlcA	Ara	Rha	Fuc	GalA	Total
LB-LNPs (No laccase)	No hem	0.02 d	0.16 d	1.44 abc	0.59 e	n.d.	0.33 bc	0.00 c	0.19 def	0.20 ab	**2.93 g**
	sdGGM	1.51 c	0.54 c	1.53 abc	0.59 e	n.d.	0.29 c	0.00 c	0.28 cdef	0.12 ab	**4.86 def**
	epGGM	2.13 b	0.64 bc	1.80 a	0.63 e	n.d.	0.31 c	0.00 c	0.22 def	0.25 ab	**5.97 bcd**
	epBLN-GGM	1.31 c	0.53 c	1.39 abc	0.54 e	n.d.	0.22 c	0.00 c	0.01 f	0.14 ab	**4.13 efg**
	sdGX	0.12 d	0.17 d	1.30 abc	2.49 d	n.d.	0.28 c	0.00 c	0.17 ef	0.28 ab	**4.82 def**
	epGX	0.13 d	0.23 d	1.30 abc	3.12 c	n.d.	0.24 c	0.00 c	0.14 af	0.29 a	**5.45 cde**
LB-LNPs (DsLcc4)	No hem	0.26 d	0.13 d	1.26 bc	0.68 e	n.d.	0.56 a	0.00 c	0.74 a	0.00 b	**3.63 fg**
	sdGGM	2.48 b	0.68 abc	1.42 abc	0.71 e	n.d.	0.48 a	0.00 c	0.61 ab	0.09 ab	**6.47 abcd**
	epGGM	3.01 a	0.76 ab	1.62 abc	0.76 e	n.d.	0.49 a	0.00 c	0.54 abc	0.09 ab	**7.27 ab**
	epBLN-GGM	3.31 a	0.84 a	1.72 ab	0.69 e	n.d.	0.48 a	0.00 c	0.46 bcd	0.00 b	**7.49 ab**
	sdGX	0.25 d	0.17 d	1.16 c	4.08 b	n.d.	0.45 a	0.07 b	0.45 bcde	0.26 ab	**6.88 abc**
	epGX	0.27 d	0.21 d	1.20 bc	4.94 a	n.d.	0.45 ab	0.13 a	0.50 abc	0.25 ab	**7.94 a**
PB-LNPs (No laccase)	No hem	0.00 e	0.00 d	0.11 ef	0.53 d	n.d.	0.04 bcde	n.d.	0.19 bc	n.d.	**0.87 f**
	sdGGM	1.04 c	0.46 a	0.31 b	0.61 d	n.d.	0.02 cde	n.d.	0.20 bc	n.d.	**2.65 d**
	epGGM	1.19 bc	0.50 a	0.36 a	0.67 d	n.d.	0.00 de	n.d.	0.23 abc	n.d.	**2.95 bcd**
	epBLN-GGM	0.58 d	0.38 b	0.25 c	0.52 d	n.d.	0.02 cde	n.d.	0.07 c	n.d.	**1.82 e**
	sdGX	0.06 e	0.19 c	0.15 d	2.01 c	n.d.	0.00 e	n.d.	0.16 bc	n.d.	**2.56 d**
	epGX	0.07 e	0.19 c	0.16 d	2.55 b	n.d.	0.03 bcde	n.d.	0.31 ab	n.d.	**3.30 bc**
PB-LNPs (DsLcc4)	No hem	0.13 e	0.16 c	0.10 f	0.51 d	n.d.	0.09 abc	n.d.	0.32 ab	n.d.	**1.31 f**
	sdGGM	1.31 ab	0.46 b	0.32 b	0.57 d	n.d.	0.12 a	n.d.	0.42 a	n.d.	**3.20 bc**
	epGGM	1.50 a	0.50 a	0.38 a	0.59 d	n.d.	0.11 ab	n.d.	0.33 ab	n.d.	**3.42 ab**
	epBLN-GGM	1.25 bc	0.47 a	0.34 ab	0.51 d	n.d.	0.13 a	n.d.	0.21 bc	n.d.	**2.91 cd**
	sdGX	0.15 e	0.18 c	0.14 de	2.50 b	n.d.	0.08 abcd	n.d.	0.27 abc	n.d.	**3.31 bc**
	epGX	0.14 e	0.22 c	0.14 de	3.04 a	n.d.	0.08 abcd	n.d.	0.24 abc	n.d.	**3.87 a**

Table S6 (Cont.). Monosaccharide composition of LNPs coated with the different hemicelluloses (*i.e.*, sdGGM, epGGM, epBLN-GGM, sdGX, and epGX) by spontaneous adsorption or *Ds*Lcc4 treatment.

LNPs	Hemicellulose coating	Monosaccharides abundance (%)									
		Man	Glc	Gal	Xyl	GlcA	Ara	Rha	Fuc	GalA	Total
BB-LNPs (No laccase)	No hem	0.00 e	0.05 c	0.00 e	1.10 de	0.22 a	n.d.	n.d.	0.13 abc	0.09 a	**1.59 e**
	sdGGM	1.16 c	0.37 ab	0.24 b	1.14 d	0.18 ab	n.d.	n.d.	0.12 abc	0.00 b	**3.19 bcd**
	epGGM	1.34 b	0.39 ab	0.30 a	1.06 de	0.18 ab	n.d.	n.d.	0.07 abc	0.03 ab	**3.36 bc**
	epBLN-GGM	0.78 d	0.27 abc	0.19 c	1.13 de	0.18 ab	n.d.	n.d.	0.04 bc	0.04 ab	**2.63 d**
	sdGX	0.10 e	0.08 c	0.06 d	2.61 c	0.18 ab	n.d.	n.d.	0.03 c	0.02 ab	**3.08 bcd**
	epGX	0.13 e	0.22 abc	0.07 d	3.00 b	0.18 ab	n.d.	n.d.	0.10 abc	0.00 b	**3.70 b**
BB-LNPs (DsLcc4)	No hem	0.11 e	0.05 c	0.00 e	0.92 de	0.16 ab	n.d.	n.d.	0.23 a	0.00 b	**1.47 e**
	sdGGM	1.36 b	0.34 ab	0.26 b	0.85 e	0.00 c	n.d.	n.d.	0.14 abc	0.00 b	**2.95 cd**
	epGGM	1.58 a	0.37 ab	0.32 a	0.87 de	0.07 bc	n.d.	n.d.	0.15 abc	0.00 b	**3.36 bc**
	epBLN-GGM	1.37 b	0.39 a	0.30 a	0.88 de	0.16 ab	n.d.	n.d.	0.22 ab	0.00 b	**3.31 bc**
	sdGX	0.10 e	0.09 c	0.08 d	2.89 bc	0.14 ab	n.d.	n.d.	0.21 ab	0.00 b	**3.51 bc**
	epGX	0.13 e	0.14 bc	0.08 d	3.75 a	0.16 ab	n.d.	n.d.	0.13 abc	0.00 b	**4.38 a**

Notes: For the same lignin type (treated with or without laccase) the averages in the column followed by the same letter do not differ from each other at a probability level of 5% according to Tukey's test. **Abbreviations:** arabinose (Ara), fucose (Fuc), galactose (Gal), galacturonic acid (GalA), glucose (Glc), glucuronic acid (GlcA), mannose (Man), not detected (n.d.), rhamnose (Rha), and xylose (Xyl).

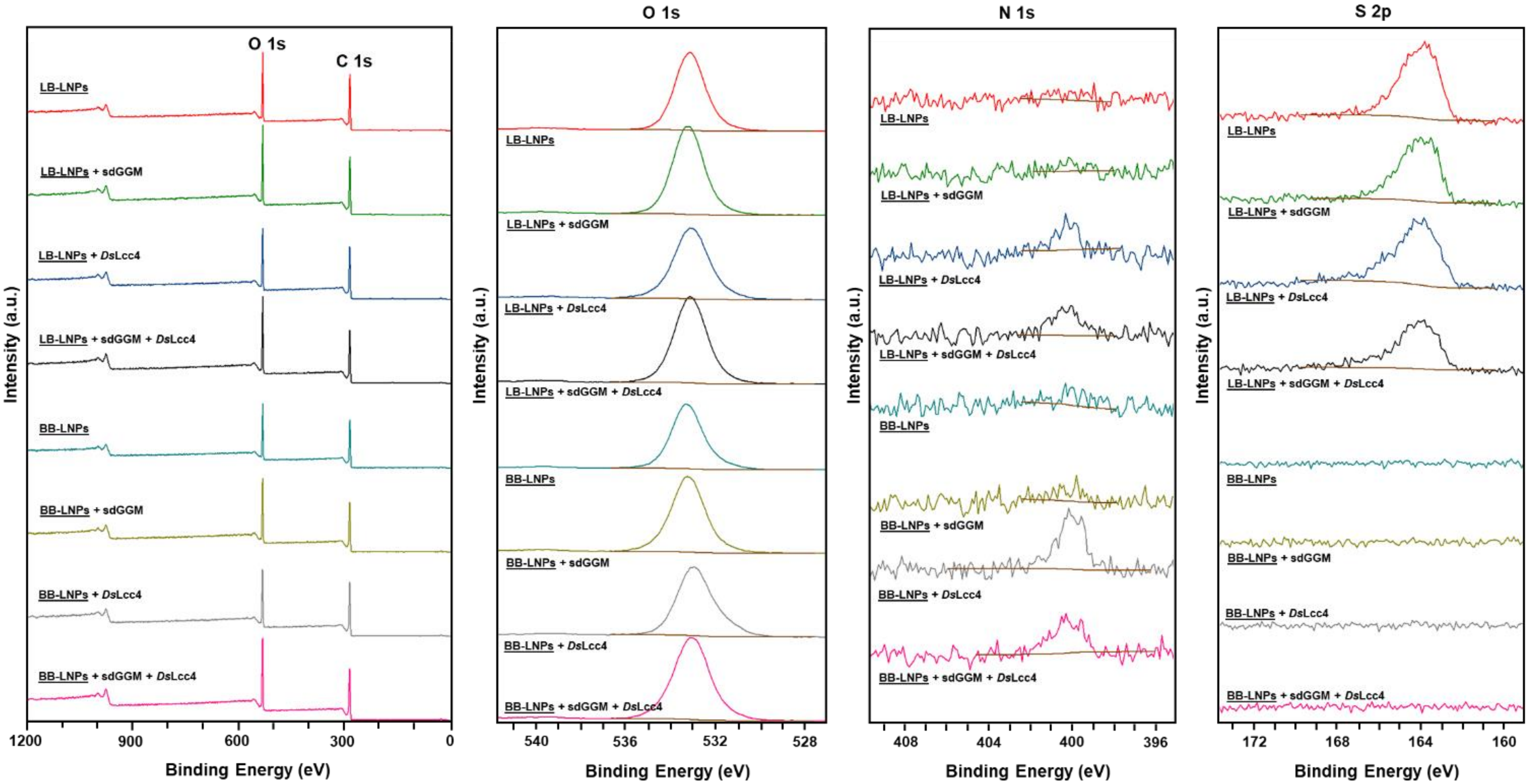

Figure S12. Wide-range survey scan (0-1200 eV) and respective high-resolution O 1s, N 1s and S 2p spectra of LB- and BB-LNP functionalized with sdGGM (2.5 mg mL^{-1}) by spontaneous absorption (no laccase), and treated with DsLcc4 (1000 nKat g^{-1} of lignin), at pH 5 and RT for 24 h. Control samples were also analyzed in the absence of laccase and/or hemicellulose.

Table S7. Elemental composition determined by X-ray photoelectron spectroscopy (XPS) of LB- and BB-LNP functionalized with sdGGM (2.5 mg mL^{-1}) by spontaneous absorption (no laccase), and treated with *Ds*Lcc4 (1000 nKat g^{-1} of lignin), at pH 5 and RT for 24 h. Control samples were also analyzed in the absence of laccase and/or hemicellulose (n = 3).

	Sample	Elemental Composition					Area of C 1s (%)		
		C 1s %	N 1s %	O 1s %	S 2p %	O/C ratio	C1 (284.7 eV)	C2 (286.4 eV)	C3 (288.1 eV)
**LB-LNPs**	**Control**	76.65 ± 0.06	0.05 ± 0.04	22.51 ± 0.07	0.79 ± 0.04	**0.294 ± 0.001**	55.3 ± 1.1	44.7 ± 1.1	
	**sdGGM (adsorption)**	75.10 ± 0.05	0.08 ± 0.05	24.19 ± 0.13	0.63 ± 0.03	**0.322 ± 0.002**	48.3 ± 1.5	51.7 ± 1.5	
	***Ds*Lcc4**	75.85 ± 0.13	0.27 ± 0.06	23.10 ± 0.09	0.79 ± 0.03	**0.305 ± 0.001**	34.8 ± 3.2	59.9 ± 7.0	5.3 ± 3.8
	**sdGGM + *Ds*Lcc4**	73.84 ± 0.27	0.21 ± 0.08	25.41 ± 0.19	0.54 ± 0.04	**0.344 ± 0.003**	43.8 ± 1.3	55.4 ± 2.3	0.8 ± 1.1
**BB-LNPs**	**Control**	77.31 ± 0.21	0.12 ± 0.05	22.57 ± 0.25	0.00 ± 0.00	**0.292 ± 0.003**	50.6 ± 0.5	49.4 ± 0.5	
	**sdGGM (adsorption)**	75.35 ± 0.05	0.18 ± 0.04	24.47 ± 0.05	0.00 ± 0.00	**0.325 ± 0.001**	42.1 ± 1.9	57.9 ± 1.9	
	***Ds*Lcc4**	75.91 ± 0.12	0.52 ± 0.01	23.58 ± 0.12	0.00 ± 0.00	**0.311 ± 0.003**	40.2 ± 1.6	53.4 ± 1.1	6.3 ± 2.1
	**sdGGM + *Ds*Lcc4**	73.78 ± 0.14	0.37 ± 0.04	25.86 ± 0.11	0.00 ± 0.00	**0.350 ± 0.002**	32.0 ± 3.3	59.4 ± 2.7	8.6 ± 1.2
